# Stabilization Of The CN_3_^5−^ Anion In Recoverable High‐pressure Ln_3_O_2_(CN_3_) (Ln=La, Eu, Gd, Tb, Ho, Yb) Oxoguanidinates

**DOI:** 10.1002/anie.202311516

**Published:** 2023-10-16

**Authors:** Andrey Aslandukov, Pascal L. Jurzick, Maxim Bykov, Alena Aslandukova, Artem Chanyshev, Dominique Laniel, Yuqing Yin, Fariia I. Akbar, Saiana Khandarkhaeva, Timofey Fedotenko, Konstantin Glazyrin, Stella Chariton, Vitali Prakapenka, Fabrice Wilhelm, Andrei Rogalev, Davide Comboni, Michael Hanfland, Natalia Dubrovinskaia, Leonid Dubrovinsky

**Affiliations:** ^1^ Bayerisches Geoinstitut University of Bayreuth Universitätstrasse 30 95440 Bayreuth Germany; ^2^ Material Physics and Technology at Extreme Conditions Laboratory of Crystallography University of Bayreuth Universitätstrasse 30 95440 Bayreuth Germany; ^3^ Institute of Inorganic Chemistry University of Cologne Greinstrasse 6 50939 Cologne Germany; ^4^ Centre for Science at Extreme Conditions and School of Physics and Astronomy University of Edinburgh EH9 3FD Edinburgh United Kingdom; ^5^ Deutsches Elektronen-Synchrotron DESY Notkestrasse 85 22607 Hamburg Germany; ^6^ Center for Advanced Radiation Sources University of Chicago Chicago Illinois 60637 USA; ^7^ European Synchrotron Radiation Facility BP 220 38043 Grenoble Cedex France; ^8^ Department of Physics Chemistry and Biology (IFM) Linköping University SE-581 83 Linköping Sweden

**Keywords:** Diamond Anvil Cell, Guanidinate, High Pressure, Lanthanides, Single-Crystal X-Ray Diffraction

## Abstract

A series of isostructural Ln_3_O_2_(CN_3_) (Ln=La, Eu, Gd, Tb, Ho, Yb) oxoguanidinates was synthesized under high‐pressure (25–54 GPa) high‐temperature (2000–3000 K) conditions in laser‐heated diamond anvil cells. The crystal structure of this novel class of compounds was determined via synchrotron single‐crystal X‐ray diffraction (SCXRD) as well as corroborated by X‐ray absorption near edge structure (XANES) measurements and density functional theory (DFT) calculations. The Ln_3_O_2_(CN_3_) solids are composed of the hitherto unknown CN_3_^5−^ guanidinate anion—deprotonated guanidine. Changes in unit cell volumes and compressibility of Ln_3_O_2_(CN_3_) (Ln=La, Eu, Gd, Tb, Ho, Yb) compounds are found to be dictated by the lanthanide contraction phenomenon. Decompression experiments show that Ln_3_O_2_(CN_3_) compounds are recoverable to ambient conditions. The stabilization of the CN_3_^5−^ guanidinate anion at ambient conditions provides new opportunities in inorganic and organic synthetic chemistry.

## Graphical abstract

A series of recoverable Ln_3_O_2_(CN_3_) (Ln=La, Eu, Gd, Tb, Ho, Yb) oxoguanidinates was synthesized under high‐pressure (25–54 GPa) high‐temperature (2000–3000 K) conditions in laser‐heated diamond anvil cells. The first stabilization of CN_3_^5−^ guanidinate anion—deprotonated guanidine—was achieved.

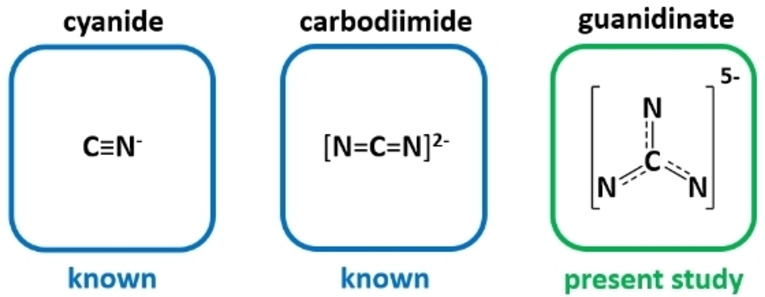

Inorganic ternary metal‐C−N compounds with covalently bonded C−N anions encompass important classes of solids. The most investigated classes are cyanides (CN^−^)[^1^, ^2^] and carbodiimides (NCN^2−^),[^3^, ^4^, ^5^, ^6^, ^7^, ^8^, ^9^] although more complex anions (i.e. dicyanamides,[^10^, ^11^] tricyanomethanides,^[12]^ and acetonitriletriide^[13]^) are known. Inorganic cyanides have applications in gold mining, metal finishing, electroplating^[14]^ and also can be used as reactants or/and catalysts in organic syntheses.^[15]^ Inorganic carbodiimides exhibit interesting optical properties, making them useful in optoelectronics and photonics[^3^, ^4^, ^5^] and have a potential for energy storage and conversion, e.g. as electrode materials for lithium‐ion batteries, fuel cells, and supercapacitors.[^6^, ^7^, ^8^, ^9^] While CN^−^ and CN_2_^2−^ anions are well‐known, the next members of this anionic series—i.e. the CN_3_^5−^ anion, a derivative of guanidine, and CN_4_^8−^, a derivative of hypothetical tetraaminomethane—have not been discovered yet, although discussed in the literature.[^16^, ^17^]

Here, we present the first stabilization of the CN_3_^5−^ guanidinate anion in the Ln_3_O_2_(CN_3_) (Ln=La, Eu, Gd, Tb, Ho, Yb) family of compounds. Unexpectedly, these compounds were discovered while studying the polynitride chemistry of rare earth elements. A series of Ln_3_O_2_(CN_3_) oxoguanidinates were synthesized at pressures and temperatures of 25 to 54 GPa and 2000–3000 K, respectively, from molecular nitrogen and metals (La, Gd, Tb, Ho) contaminated by oxygen, as well as from O‐contaminated azides Eu(N_3_)_2_ and Yb(N_3_)_2_ in a laser‐heated diamond anvil cell (DAC). The crystal structures of Ln_3_O_2_(CN_3_) oxoguanidinates were determined and refined on the basis of single‐crystal X‐ray diffraction (SCXRD) and corroborated by X‐ray absorption near edge structure (XANES) measurements and by density functional theory (DFT) calculations. The family of Ln_3_O_2_(CN_3_) compounds and CN_3_^5−^ anion were found to be quenchable to ambient conditions.

Among all synthesized compounds, the best SCXRD data quality was obtained for La_3_O_2_(CN_3_), therefore we will first discuss the crystal structure of La_3_O_2_(CN_3_) and its evolution on decompression and then discuss other Ln_3_O_2_(CN_3_) (Ln=Eu, Gd, Tb, Ho, Yb) family members and the trends in their crystal‐chemistry.

For the high‐pressure high‐temperature synthesis of the La_3_O_2_(CN_3_) compound, pieces of lanthanum were loaded in air (leading to the partial oxidization of the metal) into the sample chamber of DACs, the latter then were gas‐loaded with molecular nitrogen (see Methods section for details). In two independent experiments, the samples were compressed to 54(1) and 25(1) GPa and laser‐heated to 2500(200) K. According to synchrotron SCXRD data, in both experiments the same novel solid, adopting an unexpected La_3_O_2_(CN_3_) composition, was synthesized, and its crystal structure was determined. It was found to have the orthorhombic space group *Pnma* (#62) and lattice parameters *a*=9.2875(7) Å, *b*=6.8259(5) Å, *c*=6.149(2) Å at 54(1) GPa and *a*=9.795(2) Å, *b*=7.0062(11) Å, *c*=6.3699(11) Å at 25(1) GPa (see Tables S1–S2 and the CIFs for the full crystallographic data^[18]^). The carbon atom in La_3_O_2_(CN_3_) comes from the diamond anvil; the latter being able to act as a carbon source and participating in the chemical reactions is well known.[^19^, ^20^, ^21^]

After the La_3_O_2_(CN_3_) synthesis at 54(1) GPa, the sample was decompressed in a few pressure steps down to ambient pressure, when the DAC was opened in air. SCXRD reflections from La_3_O_2_(CN_3_) crystallites could be traced down to ambient conditions and persisted after air exposure (Table S3 and CIF for the full crystallographic data^[18]^). Thus, La_3_O_2_(CN_3_) is recoverable to ambient conditions and, at least for some time, resistant to atmospheric oxygen and moisture. One can assume that the presence of O^2−^ anions in the phase plays an important role in its stability at ambient conditions.

Although it is often difficult to distinguish C/N/O atoms in the crystal structure from the SCXRD data, in the present study it was successfully done based on the value of the R_1_ agreement factor, the ADPs ratio, interatomic distances, and the charge balance. A detailed justification of the structure model is provided in Supplementary Discussion 1 of the Supporting Information. The structure model was confirmed by supporting DFT calculations using the Vienna ab initio simulation package.^[22]^ DFT calculations show that the relaxed structural parameters for La_3_O_2_(CN_3_) (Table S4) closely reproduce the corresponding experimental values at 54(1) GPa, as well as at 1 bar. Phonon dispersion relations calculated in the harmonic approximation show that the La_3_O_2_(CN_3_) phase is dynamically stable at 54 GPa (Figure S1a), as well as at ambient pressure (Figure S1b).

Under high pressure, the elements can behave differently than under ambient conditions, featuring exotic chemistry and unusual oxidation states.[^23^, ^24^, ^25^] Therefore, in this study, to be sure in charge balance considerations, the +3 oxidation state of lanthanum in La_3_O_2_(CN_3_) was confirmed by synchrotron La *L*_II_ edge XANES measurements at the ID12 beamline at ESRF. The position of the lanthanum white line in La_3_O_2_(CN_3_) matches the white line position of the reference sample (La_2_O_3_), indicating the same +3 oxidation state of La in these compounds (Figure 1). The structure of La_3_O_2_(CN_3_) (Figure 2a–c) has two La, one C, two N, and two O distinct crystallographic atomic positions. Carbon and nitrogen atoms form planar trigonal CN_3_ units (Figure 2d, e), while both oxygen atoms are isolated distinct atoms. O1 atoms are surrounded by six La atoms at the apexes of a distorted octahedron. The O1La_6_ octahedra are interconnected with each other by common vertices, forming a three‐dimensional framework, whereas the CN_3_ triangles and isolated O2 atoms are positioned in the voids between the octahedra (Figure 2b, c).

**Figure 1:**
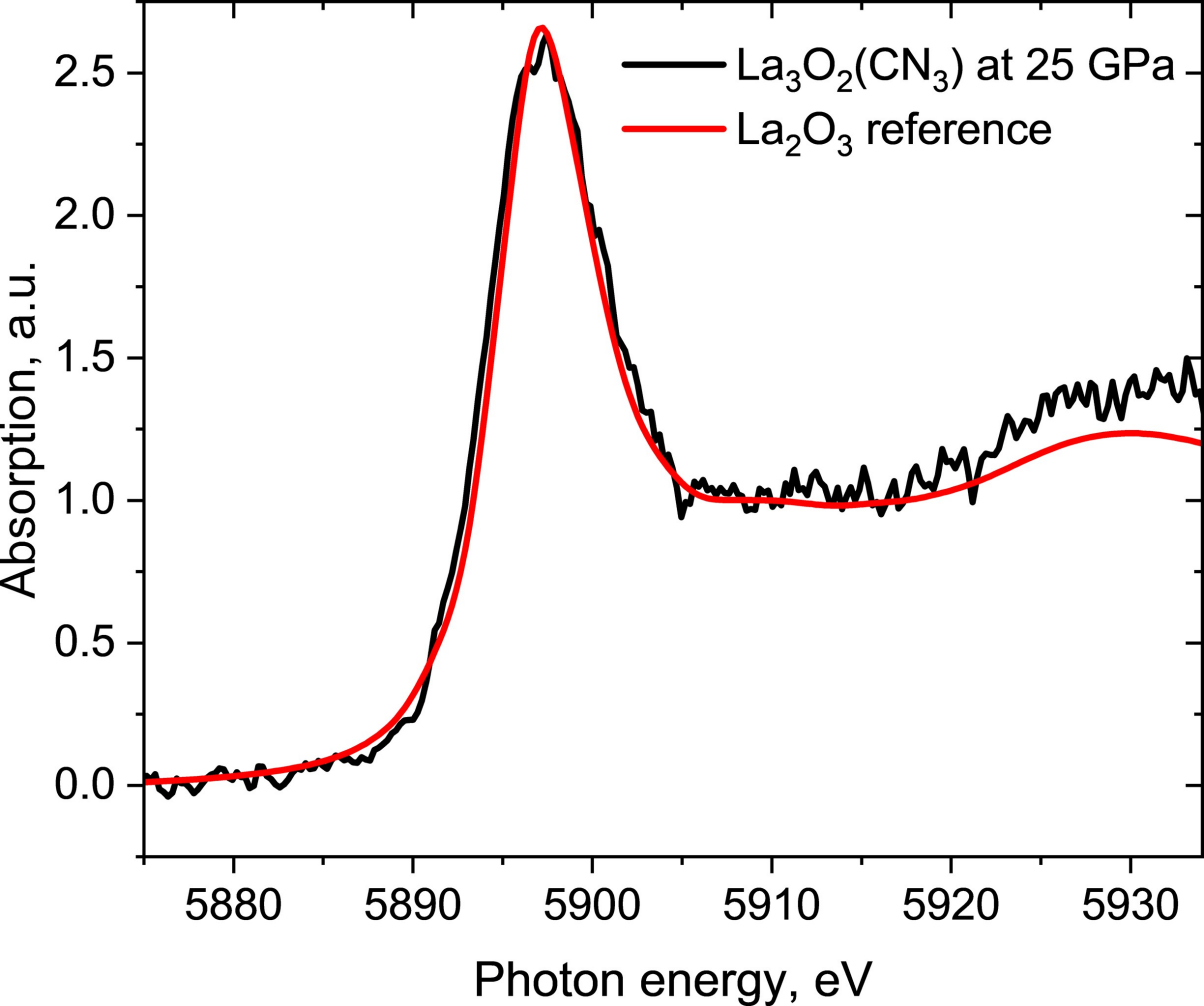
La *L*_II_ edge XANES spectra of La_3_O_2_(CN_3_) sample at 25 GPa and La_2_O_3_ reference sample at ambient conditions. The position of white lines is 5897 eV in both spectra. The low signal‐to‐noise ratio in the spectrum of La_3_O_2_(CN_3_) is because the measurements were carried out in a DAC, and 2 mm‐thick diamonds absorb 98 % of the X‐ray radiation at these energies.

**Figure 2:**
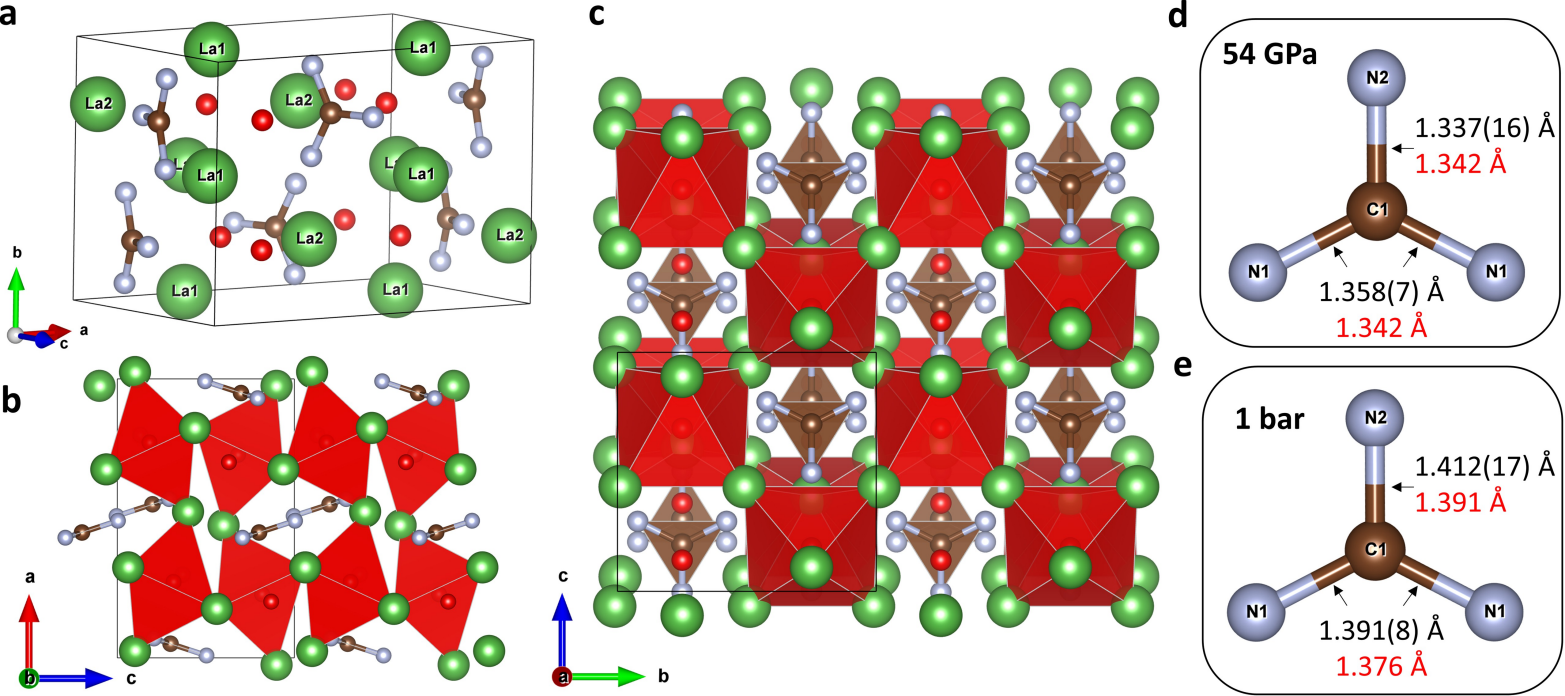
Crystal structure of La_3_O_2_(CN_3_). (a) A general view. (b) A view along the *b*‐axis. (c) A view along the *a*‐axis. (d) CN_3_ unit at 54 GPa. (e) CN_3_ unit at 1 bar. La atoms are green, C atoms are brown, N atoms are gray, and O atoms are red. Red distorted octahedra correspond to O1La_6_. Thin grey lines outline the unit cell. The bond length values obtained from experiments are shown in black, while those obtained from the DFT calculations are shown in red.

La1 atoms are coordinated by five N atoms and four O atoms (the coordination number CN=9, the coordination polyhedron is a distorted capped square antiprism), while La2 atoms are eight‐fold coordinated by four N atoms and four O atoms (coordination number CN=8, the coordination polyhedron is irregular) (Figure S2).

Compared to the HNC(NH_2_)_2_ guanidine molecule, which has two types of C−N bonds: two single C−N bonds (d_C−N_=1.36 Å) and one double C=N bond (d_C−N_=1.30 Å), the deprotonated CN_3_^5−^ unit (Figure 2d, e) in La_3_O_2_(CN_3_) has three almost equal C−N bonds indicating the delocalization of π‐bond. The average length of the C−N bond in CN_3_^5−^ at 1 bar is d_C−N_≈1.40 Å, which is longer than an average C−N distance in guanidine (d_C‐N_=1.34 Å). At the same time, the average length of the C−N bond in CN_3_^5−^ at 1 bar is between those of a typical single C−N bond (d_C−N_≈1.47 Å) and a bond of the order of 1.5 (in pyridine d_C−N_=1.35 Å), suggesting an expected bond order of 1.33 (Figure 2d). The C−N bond in CN_3_^5−^ is longer than C−O and N−O bonds in the well‐known trigonal CO_3_^2−^ carbonate (d_C‐O_=1.27–1.29 Å) and NO_3_^−^ nitrate anions (d_N‐O_=1.24–1.26 Å) at 1 bar, respectively.

There are known at ambient conditions quaternary Ln−C−N−O compounds with a stable stoichiometry Ln_2_O_2_CN_2_: *I*4*/mmm*‐La_2_O_2_CN_2_ ^[26]^ and *P*‐3*m*1‐Ln_2_O_2_(CN_2_) (Ln=Ce, Pr, Nd, Sm, Eu, Gd, Dy, Ho, Er, Tm, Yb).[^27^, ^28^] The crystal structures of Ln_2_O_2_CN_2_ compounds consist of Ln_2_O_2_^2+^ layers and linear CN_2_^2−^ anions at the interlayer positions oriented whether along^[26]^ or perpendicular[^27^, ^28^] to the layers. Thus, the structure motif of Ln_2_O_2_CN_2_ compounds completely differs from those of La_3_O_2_(CN_3_).

The crystal structure of the La_3_O_2_(CN_3_) compound has similarities with that of the La_3_(SiN_3_O)O oxonitridosilicate^[29]^ (Figure 3a, b). Both structures are based on OLa_6_ octahedra framework. While Si atoms form [SiN_3_O]^7−^ tetrahedra in La_3_(SiN_3_O)O, carbon atoms in La_3_O_2_(CN_3_) prefer a trigonal coordination, and the (CN_3_O) ensemble of atoms thereby exists as a CN_3_^5−^ unit and a distinct O^2−^ anion (Figure 3c, d).

**Figure 3:**
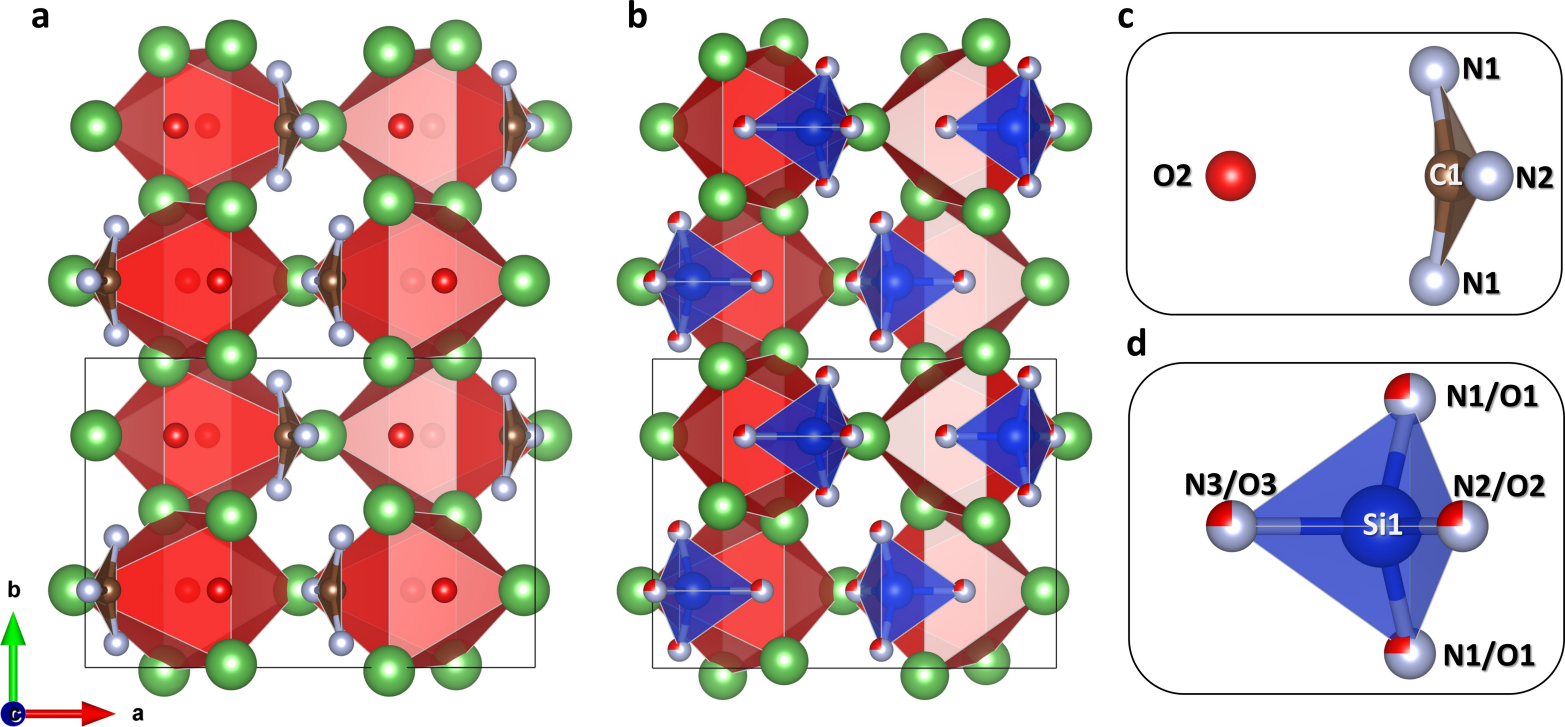
Comparison of the structures of La_3_O_2_(CN_3_) and La_3_(SiN_3_O)O oxonitridosilicate. (a) Crystal structure of La_3_O_2_(CN_3_) viewed along the *c*‐axis. (b) Crystal structure of La_3_(SiN_3_O)O^[29]^ viewed along the *c*‐axis. (c) and (d) The arrangements of CN_3_O and SiN_3_O ensembles of atoms in both compounds. La atoms are green, C atoms are brown, N atoms are gray, O atoms are red, and Si atoms are blue. Red distorted octahedra correspond to O1La_6_ octahedra. Thin grey lines outline the unit cell.

The calculated electron localization function for La_3_O_2_(CN_3_) confirms the expected covalent bonding between carbon and nitrogen atoms within the CN_3_ units (Figure 4a). There is no electron localization between C1 and O2 atoms (Figure 4b) that corroborates the absence of covalent interactions between the CN_3_ unit and the O2 atom, and thus there is no CN_3_O tetrahedron in the studied material. A reason for that important difference in the crystal chemistry of carbon and silicon is the smaller radius of carbon, which therefore prefers a lower coordination number than its group neighbor silicon at ambient conditions. It is a well‐known difference between carbonates and silicates at ambient conditions: carbon forms trigonal CO_3_^2−^ groups, while silicon prefers SiO_4_ tetrahedra. Only under sufficient compression (at least to 20 GPa) carbon starts to behave like silicon and form CO_4_^4−^ tetrahedral anions.[^30^, ^31^, ^32^, ^33^, ^34^] The recent high‐pressure high‐temperature synthesis of C_3_N_4_ polymorphs^[21]^ shows that, above 70 GPa, CN_4_ tetrahedra can also be formed. Our experimental results demonstrate that 54 GPa is not a sufficient pressure for the formation of tetra‐coordinated carbon with C−N bonds. However, one can expect the stabilization of CN_4_^8−^ units or/and the formation of polycarbonitrides built of corner/edge‐sharing CN_4_ tetrahedra in ternary M−C−N systems at pressures above 70 GPa.

**Figure 4:**
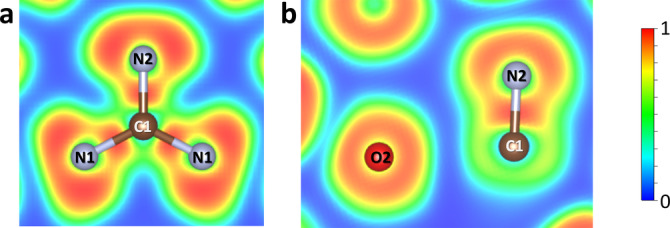
Cross sections of the electron localization function calculated for La_3_O_2_(CN_3_). (a) In the plane of a CN_3_^5−^ unit. (b) In the (0 1 0) plane containing C1, N2, and O2 atoms.

The structure evolution and compressibility of La_3_O_2_(CN_3_) were studied experimentally upon the decompression from 54(1) GPa down to 1 bar, as well as using DFT by variable‐cell structure relaxation in the pressure range of 0 to 100 GPa. The lattice parameters and unit cell volume of La_3_O_2_(CN_3_) extracted from the SCXRD data collected upon the decompression are closely reproduced by DFT (Figure 5).

**Figure 5:**
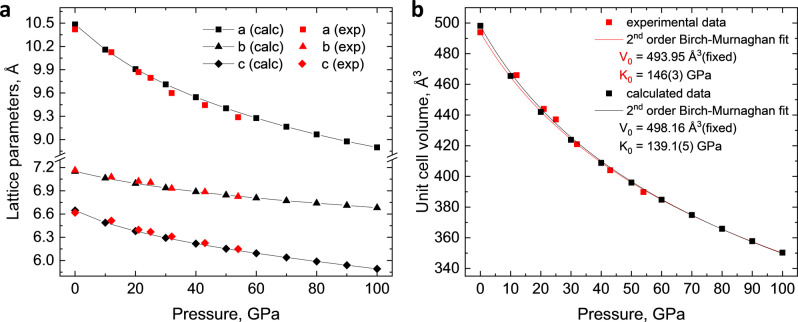
Compressional behavior of the La_3_O_2_(CN_3_) structure. (a) Experimental (red squares, triangles, and diamonds) and calculated (black squares, triangles, and diamonds) pressure dependence of the La_3_O_2_(CN_3_) lattice parameters. (b) Experimental (red squares) and calculated (black squares) pressure dependence of the La_3_O_2_(CN_3_) unit cell volume and the fit of the experimental P−V data (red curve) and calculated P−V data (black curve) using a 2^nd^ order Birch‐Murnaghan equation of state. Errors in the experimental data are within the symbol size. The calculated P−V data also can be fitted using the 3^rd^ order Birch‐Murnaghan equation of state, yielding K_0_=131.3(9) GPa, K′=4.36(4) with fixed V_0_=498.16 Å^3^.

The evolution of the *a*, *b*, and *c* lattice parameters (Figure 5a) shows an anisotropic response of La_3_O_2_(CN_3_) to pressure: its compressibility along the three main directions increases in the following order: *a*>*c*>*b* (it is also clear from the evolution of the *a*/*a_0_*, *b*/*b_0_*, *c*/*c_0_* ratio with pressure, Figure S3). One can explain the greater compressibility along the *a* direction due to the larger spacing between O1La_6_ octahedra in that direction (Figure 1b). The coordination numbers and coordination polyhedra of La1 and La2 atoms do not change in the studied pressure range.

The fitting of the La_3_O_2_(CN_3_) experimental pressure‐volume data with a 2^nd^ order Birch‐Murnaghan equation of state yields a bulk modulus of *K*_0_=146(3) GPa (*V*_0_=493.95 Å^3^ was fixed), a value that agrees well with the bulk modulus *K*_0_=139.1(5) GPa (*V*_0_=498.16 Å^3^ was fixed) obtained by fitting the pressure‐volume points of La_3_O_2_(CN_3_) DFT‐relaxed structures from 0 to100 GPa with the 2^nd^ order Birch‐Murnaghan equation of state (Figure 5b).

The synthesis of the other Ln_3_O_2_(CN_3_) (Ln=Eu, Gd, Tb, Ho, Yb) family members was done under similar high‐pressure high‐temperature conditions (pressures of 25 to 54 GPa and temperatures of 2000–3000 K) from partially oxidized metals (Gd, Tb, Ho) and nitrogen as well as from oxygen‐contaminated azides Eu(N_3_)_2_ and Yb(N_3_)_2_ in a laser‐heated DAC (Table S1). The crystal structures of Ln_3_O_2_(CN_3_) (Ln=Eu, Gd, Tb, Ho, Yb) oxoguanidinates were also determined based on SCXRD (Tables S6–S13). The fact that Ln_3_O_2_(CN_3_) phases were obtained for different lanthanides at different pressures and from different precursors demonstrates the stability of such a structure type and chemical composition.

It should be noted that contrary to the case of La_3_O_2_(CN_3_), the crystal structure refinements for Ln_3_O_2_(CN_3_) (Ln=Eu, Gd, Tb, Ho, Yb) indicate a possible splitting of the C1 atom between two crystallographic positions (see Supplementary Discussion 2). While models with split C1 atom position resulted in a slightly lower R_1_ agreement factor, we cannot exclude that this is an artifact of the limited quality of X‐ray diffraction data collected in the DACs. The investigation of this phenomenon requires additional studies. Here we will consider the structure models without splitting for all Ln_3_O_2_(CN_3_) (Ln=Eu, Gd, Tb, Ho, Yb) compounds.

The structural evolution of these compounds with pressure was also studied by DFT calculations (Figures S4–S8). For Tb_3_O_2_(CN_3_) and Ho_3_O_2_(CN_3_), the decompression down to 1 bar was done, and the experimentally observed P−V dependence is well‐reproduced by DFT calculations (Figs. S6 and S7). For Ln=Gd, Tb, Ho the recoverability of the synthesized compounds was examined, and they were found to be recoverable, just like La_3_O_2_(CN_3_) (Tables S7, S10, and S12). The volume of the Ln_3_O_2_(CN_3_) unit cell at the same pressure decreases when going through the sequence La−Eu−Gd−Tb−Ho−Yb, as expected due to the lanthanide contraction (Figure 6a). Moreover, the correlation between the unit cell volume and ionic radii^3^ is linear, which is similar to known classes of lanthanide compounds (e.g. nitrides, Figure S9).

**Figure 6:**
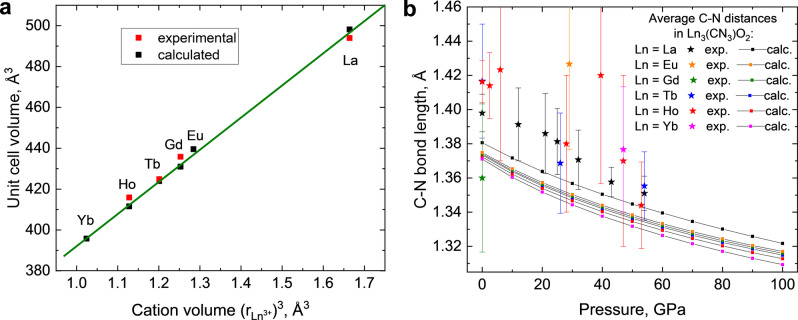
Comparison of crystallographic characteristics of the studied Ln_3_O_2_(CN_3_) compounds at 1 bar and in the range up to 100 GPa. (a) The volume of the Ln_3_O_2_(CN_3_) unit cell at 1 bar versus the volume of lanthanide ion (ionic radii are taken from http://abulafia.mt.ic.ac.uk/shannon/ptable.php (original reference^[35]^) for CN=8). (b) The average C−N bond length within the CN_3_^5−^ unit in Ln_3_O_2_(CN_3_) (Ln=La, Eu, Gd, Tb, Ho, Yb) at different pressures.

The behavior of the CN_3_^5−^ anion under compression was investigated both experimentally and theoretically for all Ln_3_O_2_(CN_3_) (Ln=La, Eu, Gd, Tb, Ho, Yb) phases (Figure 6b). Due to significant uncertainties in C−N distances extracted from the experimental data, no remarkable differences in the C−N bond lengths dependent on a lanthanide cation at the same pressure was noted, but there is a general trend of a small decrease of the C−N bond length upon compression. The analysis of theoretical data provides a more resolved picture: regardless of a cation, C‐N bonds become shorter by ≈0.04 Å upon compression from 1 bar to 100 GPa, and furthermore, a small and monotonous contraction of the C−N bond length in CN_3_^5−^ is noted when going from lanthanum to ytterbium. At the same time, metal‐nonmetal distances change significantly depending on both the pressure and the cation (Figure S10), indicating that the decrease in the unit cell volume is primarily attributed to the decrease in the volume of the Ln coordination polyhedra. Due to this reason, the calculated bulk moduli of Ln_3_O_2_(CN_3_) solids decrease along the Ln=La−Eu−Gd−Tb−Ho−Yb lanthanide row (Table S14, Figure S11).

To conclude, a series of recoverable Ln_3_O_2_(CN_3_) (Ln=La, Eu, Gd, Tb, Ho, Yb) oxoguanidinates was synthesized under high‐pressure high‐temperature conditions. The fact that the Ln_3_O_2_(CN_3_) phases were obtained for different lanthanides, under different pressures, and from different precursors, demonstrates the stability of such a structure type and chemical composition. Despite the significant difference in ionic radii of La^3+^ and Yb^3+^, all studied Ln_3_O_2_(CN_3_) compounds are isostructural and the only difference in their crystal chemistry is the unit cell volumes and interatomic distances decreasing due to the lanthanides’ contraction. The lanthanide contraction also dictates the slight decrease in compressibility of Ln_3_O_2_(CN_3_) compounds going from Ln=La to Ln=Yb. These solids all feature the hitherto unknown CN_3_^5−^ guanidinate anion—i.e. deprotonated guanidine. This discovery extends the list of carbon‐nitrogen inorganic anions and may open up new synthesis routes in inorganic and organic chemistry. Also, based on the analysis of carbon crystal‐chemical behavior under pressure and its comparison with silicon crystal chemistry, one can expect the stabilization of currently unknown CN_4_^8−^ unit or/and formation of polycarbonitrides built of corner/edge‐sharing CN_4_ tetrahedra in ternary M−C−N systems at pressures above 70 GPa.

*Note*: At the same time, the high‐pressure synthesis of SbCN_3_ antimony nitridocarbonate was carried out by another group,^[36]^ which demonstrates the universality of a high‐pressure approach for the stabilization of CN_3_^5−^ guanidinate anion.

## Conflict of interest

The authors declare no conflict of interest.

## Supporting information

As a service to our authors and readers, this journal provides supporting information supplied by the authors. Such materials are peer reviewed and may be re‐organized for online delivery, but are not copy‐edited or typeset. Technical support issues arising from supporting information (other than missing files) should be addressed to the authors.

Supporting Information

Supporting Information

Supporting Information

Supporting Information

## Data Availability

The data that support the findings of this study are available in the supplementary material of this article. Additional references are cited within the Supporting Information.[^37^, ^38^, ^39^, ^40^, ^41^, ^42^, ^43^, ^44^, ^45^, ^46^, ^47^, ^48^, ^49^, ^50^, ^51^, ^52^, ^53^, ^54^, ^55^, ^56^, ^57^, ^58^, ^59^, ^60^]
