# Supplementary material for: Stabilization Of The CN3 5− Anion In Recoverable High‐pressure Ln3O2(CN3) (Ln=La, Eu, Gd, Tb, Ho, Yb) Oxoguanidinates
Source: Angew Chem Int Ed Engl. 2023 Oct 16;62(47):e202311516. doi: 10.1002/anie.202311516 (PMC11497228; doi:10.1002/anie.202311516)
Supplement: Supplementary file 4 — Supporting Information [file ANIE-62-e202311516-s002.pdf]

Angewandte Chemie
Eine Zeitschrift der Gesellschaft Deutscher Chemiker

Supporting Information

Stabilization Of The CN_3^{5-} Anion In Recoverable High-pressure $Ln_3O_2(CN_3)$ (Ln = La, Eu, Gd, Tb, Ho, Yb) Oxoguanidinates

A. Aslandukov, P. L. Jurzick, M. Bykov, A. Aslandukova, A. Chanyshev, D. Laniel, Y. Yin, F. I. Akbar, S. Khandarkhaeva, T. Fedotenko, K. Glazyrin, S. Chariton, V. Prakapenka, F. Wilhelm, A. Rogalev, D. Comboni, M. Hanfland, N. Dubrovinskaia, L. Dubrovinsky*

Table of Contents

Methods 2
Supplementary Discussion 1. The justification of the selected $La_3O_2(CN_3)$ structure model. 4
Supplementary Discussion 2. Possible splitting of C1 atom position in $Ln_3O_2(CN_3)$ (Ln = Eu, Gd, Tb, Ho, Yb) structures. 6
Supplementary Figures 7
Supplementary Tables 12
References 25

Methods

Sample preparation. The BX90-type large X-ray aperture diamond anvil cells[1] equipped with Boehler-Almax type diamonds[2] (culet diameter is 250 μm) were used in the experiments. The sample chamber was formed by pre-indenting a rhenium gasket to 20-25 μm thickness and drilling a hole of 110-125 μm in diameter in the center of the indentation. The pieces of lanthanides (La, Gd, Tb, Ho) (99.9%, ChemPur or smart-elements) were placed in the sample chambers of individual DACs in air, then molecular nitrogen was loaded using a high-pressure gas loading system (1300 bars) [3]. The metal pieces were exposed to air during the loading into DAC. The exposure time depends on the loading but always was between 20 min and 1 hour. The investigation of the metal pieces exposed to air for 20 min by powder XRD at a home diffractometer showed that corresponding rare-earth oxides were detectable in the powder pattern at the level of ~3-5wt%. This is an estimation of minimal contamination by oxides. The sample chambers of another two DACs were loaded with solid $Eu(N_3)_2$ and $Yb(N_3)_2$ compounds, whose syntheses will be described elsewhere, in the glovebox. The samples were compressed to target pressure (Table S1) and laser-heated (λ = 1064 nm) to 2000-3000 K using double-sided laser-heating systems BGI (University of Bayreuth, Bayreuth, Germany) [4,5] or of P02.2 beamline (Petra III, DESY, Hamburg, Germany). The temperature during laser heating was determined by the blackbody radiation fit. The initial pressure in the sample chambers, as well as pressure upon the decompression of some of the cells, was determined by the Raman signal from the diamond anvils[6] and additionally monitored by the diffraction of Re gasket edge using the equation of state of Re.[7]

X-ray diffraction. The X-ray diffraction studies were done at the P02.2 beamline of Petra III, DESY (λ = 0.2904 Å or 0.2905 Å depending on the experiment); ID15b beamline (λ = 0.4104 Å) of the Extreme Brilliant Source European Synchrotron Radiation Facility (EBS-ESRF); as well as at the GSECARS 13IDD beamline of the APS (λ = 0.2952 Å). At P02.2 beamline of DESY the X-ray beam was focused down to 2 × 2 μm^2 and data was collected with a PerkinElmer 1621 XRD flat-panel detector. At ID15b beamline of ESRF the X-ray beam was focused down to 3 × 3 μm^2 and data was collected with Eiger2X CdTe 9M hybrid photon counting pixel detector. At 13IDD beamline of APS the X-ray beam was focused down to 2 × 2 μm^2 and data was collected with Pilatus 1M detector. In order to determine the position of the polycrystalline sample on which the single-crystal X-ray diffraction acquisition is obtained, a full X-ray diffraction mapping of the pressure chamber was achieved. The sample position displaying the most and the strongest single-crystal reflections belonging to the phase of interest was chosen for the collection of single-crystal data, collected in step-scans of 0.5° from −36° to +36°. The CrysAlis^{Pro} software package[8] was used for the analysis of the single-crystal XRD data (peak hunting, indexing, data integration, frame scaling, and absorption correction). To calibrate an instrumental model in the CrysAlis^{Pro} software, i.e., the sample-to-detector distance, detector's origin, offsets of the goniometer angles, and rotation of both the X-ray beam and detector around the instrument axis, we used a single crystal of orthoenstatite [$(Mg_{1.93}Fe_{0.06})(Si_{1.93},Al_{0.06})O_6$, *Pbca* space group, a = 8.8117(2) Å, b = 5.18320(10) Å, and c = 18.2391(3) Å]. The DAFi program was used for the search of reflection's groups belonging to the individual single crystal domains.[9] Using the OLEX2 software package,[10] the structures were solved with the ShelXT structure solution program[11] using intrinsic phasing and refined with the ShelXL[12] refinement package using least-squares minimization. Crystal structure visualization was made with the VESTA software.[13] The equations of state were obtained by fitting the pressure-volume dependence data using the EoSFit7-GUI.[14]

XANES. The x-ray absorption near edge structure (XANES) experiments at the La L_{II} edge (~5.9 keV) were performed at the beamline ID12 of the ESRF in Grenoble. The beam was focused down to 5 × 5 μm^2 using two parabolic two-dimensional refractive beryllium lenses having a parabola apex radius of 50 μm fixing the focal distance to be at 2.0 m. The beam size has been checked to remain constant within the La L_{II}-edge energy range. The XANES spectra were recorded via the total fluorescence yield using a photodiode in backscattering geometry. The XANES spectra were collected from the $La_3O_2(CN_3)$ sample in DAC#2 at 25 GPa and from La_2O_3 reference sample at ambient conditions.

SUPPORTING INFORMATION

Theoretical Calculations. First-principles calculations were performed using the framework of density functional theory (DFT) as implemented in the Vienna Ab initio Simulation Package (VASP).[15] The Projector-Augmented-Wave (PAW) method[16] was used to expand the electronic wave function in plane waves. The Generalized Gradient Approximation (GGA) functional is used for calculating the exchange-correlation energies, as proposed by Perdew–Burke–Ernzerhof (PBE).[17] The PAW potentials "C", "N" and "O" with the following valence configurations $2s^2 2p^2$ for C, $2s^2 2p^3$ for N, $2s^2 2p^4$ for O were used for non-metal atoms. For lanthanum "La" PAW potential was used with the $5s^2 5p^6 5d^1 6s^2$ valence configuration, while for Ln = Eu, Gd, Tb, Ho, Yb the "Ln_3" PAW potentials were used, in which non-valence 4 *f*-electrons are kept frozen in the core. We used the Monkhorst–Pack scheme with 4×6×6 *k*-points for Brillouin zone sampling for all $Ln_3O_2(CN_3)$ compounds, and the plane-wave kinetic energy cutoff was set to 800 eV, with which total energies are converged to better than 2 meV/atom. The 0 K harmonic phonon calculations were performed using the finite displacement approach implemented into PHONOPY.[18] Converged phonon dispersions were achieved using a (1×2×2) supercell with 144 atoms and 6×4×4 Monkhorst-Pack k-point sampling.

Supplementary Discussion 1. The justification of the selected $La_3O_2(CN_3)$ structure model.

The crystal structure solution unambiguously suggested two positions of La atoms and five positions of C/N/O non-metal atoms (two positions of distinct atoms (X1 and X2) and three positions in an AZ_3 planar trigonal unit, in general, $La_3X1X2(AZ_3)$). The assignment of C/N/O atoms required a more detailed analysis, which is described below.

First, the atoms in the AZ_3 planar trigonal unit were determined. The well-known trigonal CO_3^{2-} carbonate and NO_3^- nitrate anions were ruled out because they did not fit the A-Z distances: $d_{(A\text{-}X)} \approx 1.35$ Å at 54 GPa and $d_{(A\text{-}X)} \approx 1.40$ Å at 1 bar, while C-O and N-O distances in CO_3^{2-} and NO_3^- ($d_{C\text{-}O}$ = 1.27-1.29 Å and $d_{N\text{-}O}$ = 1.24-1.26 Å at 1 bar) are significantly shorter. Therefore, we considered two other options: (1) an NN_3^{4-} unit, which was theoretically predicted in several studies[19–23] with $d_{N\text{-}N}$ = 1.35 Å at 40 GPa and (2) an CN_3^{5-} unit. Carbon, nitrogen and oxygen atoms were tested for X1 and X2. The results of the analysis of the crystal structure refinement are summarized in Table SD1.1. Upon preliminary analysis, the possibility of the X1 and X2 positions occupation by carbon atoms was ruled out due to much worse R_1 and ADPs values, so we do not include these models in Table SD1.1.

Table SD1.1. Analysis of the structure refinements of different "$La_3X1X2(AZ_3)$" models at 54(1) GPa.

Model $La_3X1X2(AZ_3)$	R_1, %	U_{eq} ADPs[1]					Max/min ADP ratio	Distances, Å		Charge balance[2]	
		"A"	"Z1"	"Z2"	"X1"	"X2"		"A-Z1"	"A-Z2"	cations	anions
$La_3NN(NN_3)$	2.81	N 0.027	N 0.006	N 0.009	N 0.003	N 0.005	9.0	1.35	1.35	+9	-10
$La_3NO(NN_3)$	2.78	N 0.028	N 0.006	N 0.009	N 0.003	O 0.010	9.3	1.36	1.33	+9	-9
$La_3ON(NN_3)$	2.76	N 0.025	N 0.006	N 0.008	O 0.006	N 0.005	5.0	1.36	1.35	+9	-9
$La_3OO(NN_3)$	2.66	N 0.023	N 0.006	N 0.008	O 0.006	O 0.010	3.8	1.34	1.36	+9	-8
$La_3NN(CN_3)$	2.70	C 0.016	N 0.006	N 0.008	N 0.003	N 0.005	5.3	1.35	1.35	+9	-11
$La_3NO(CN_3)$	2.65	C 0.015	N 0.006	N 0.008	N 0.003	O 0.010	5.0	1.35	1.34	+9	-10
$La_3ON(CN_3)$	2.61	C 0.013	N 0.006	N 0.007	O 0.006	N 0.005	2.6	1.35	1.36	+9	-10
$La_3OO(CN_3)$	2.53	C 0.013	N 0.006	N 0.007	O 0.006	O 0.010	2.2	1.36	1.34	+9	-9
$La_3O(O_{0.25}N_{0.75})(CN_{2.25}O_{0.75})$	2.60	C 0.013	¾N ¼O 0.007	¾N ¼O 0.009	O 0.006	¾N ¼O 0.006	2.2	1.35	1.35	+9	-9

[1]U_{eq} ADPs of both La1 and La2 were $U_{eq} \approx 0.007$ in each structure model.
[2]Charges were calculated in classic ionic considerations.
The colors represent the reliability of the analyzed parameters: green – good, orange – acceptable, and red – inadequate. The uncertainty of the U_{eq} ADPs and of the "A-Z" distances are omitted for clarity.

The A-Z bond lengths in the AZ_3 unit were found to be the same within the uncertainty for all tested structure models. All models with NN_3^{4-} units have central nitrogen atoms with relatively large ADPs, which results in a huge max/min ADPs ratio of non-metal atoms, indicating that the structure models are not correct. Models with CN_3^{5-} unit have smaller ADP values of carbon atoms, resulting in a good max/min ADPs ratio. The R_1 factor also drops down compared to structure models with NN_3^{4-} unit. The lowest R_1 factor and smallest max/min ADPs ratio were achieved with a $La_3O_2(CN_3)$ structure model. Moreover, only this composition among all models containing CN_3^{5-} units is reasonable from charge balance considerations.

For $La_3O_2(CN_3)$ composition we also tested $La_3O(O_{0.25}N_{0.75})(CN_{2.25}O_{0.75})$ model, where N1, N2, and O2 positions are mixed-occupied with N and O in an atomic ratio of ¾:¼, similar to the proposed model for $La_3(SiN_3O)O$ oxonitridosilicate.[24] This change resulted in a slight increase of R_1 factor, indicating that the mixed-occupied model is not preferable.

Table SD1.2. The results of DFT analysis of different $La_3X1X2(AZ_3)$ structure models at 1 bar

Model	Unit cell volume		Dynamical stability
	V_{exp}, Å^3	V_{calc}, Å^3	
$La_3NN(NN_3)$	493.95(9)	515.67	No
$La_3OO(NN_3)$	493.95(9)	498.92	No
$La_3NN(CN_3)$	493.95(9)	512.71	Yes
$La_3OO(CN_3)$	493.95(9)	498.16	Yes

DFT calculations additionally corroborate the validity of a $La_3O_2(CN_3)$ model. We compared the relaxed structure models of the potential compositions, at 1 bar, in terms of (1) dynamical stability, and (2) discrepancy between the experimental and calculated unit cell volumes (Table SD1.2). Phonon calculations show that all relaxed models with NN_3^{4-} unit have imaginary phonon frequencies and therefore are dynamically unstable at 1 bar (Fig. SD1.1). On the other hand, models comprised of

CN_3^{5-} units are dynamically stable (Fig. SD1.2) and the $La_3O_2(CN_3)$ composition has the best agreement between experimental and calculated unit cell volumes.

Thus, both detailed structure refinement analysis and DFT calculations unambiguously show that the selected $La_3O_2(CN_3)$ structure model is correct.

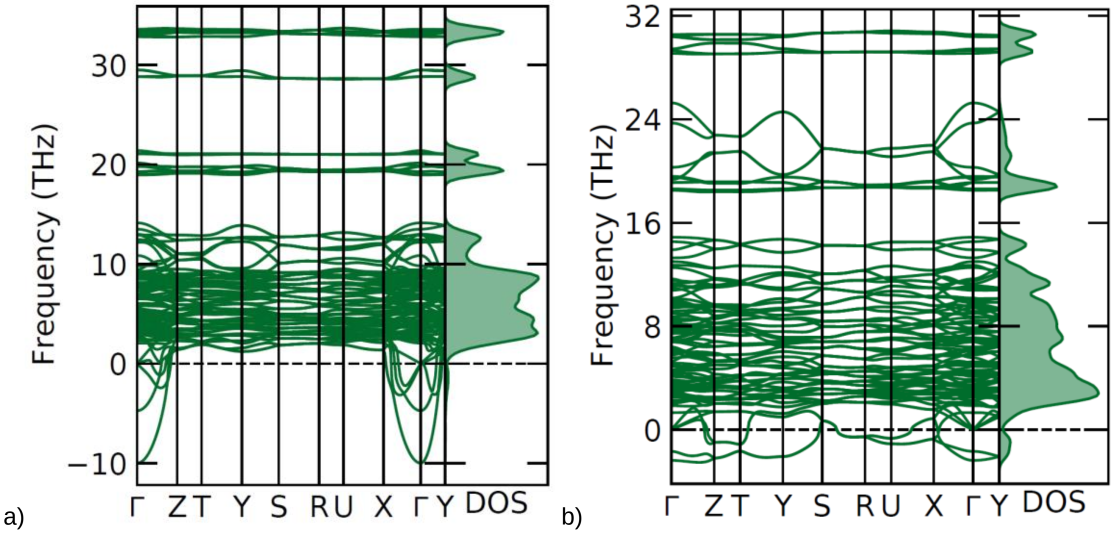

Figure SD1.1. Phonon dispersion curves for (a) $La_3N_2(NN_3)$ and (b) $La_3O_2(NN_3)$ along the high symmetry directions in the Brillouin zone and the phonon density of states at ambient pressure.

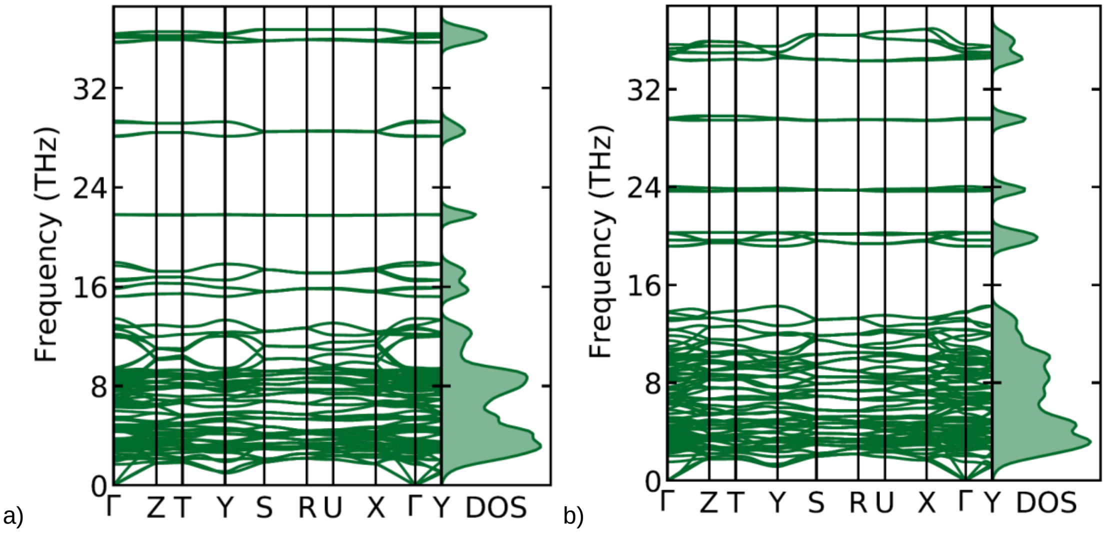

Figure SD1.2. Phonon dispersion curves for (a) $La_3N_2(CN_3)$ and (b) $La_3O_2(CN_3)$ along the high symmetry directions in the Brillouin zone and the phonon density of states at ambient pressure.

Supplementary Discussion 2. Possible splitting of C1 atom position in $Ln_3O_2(CN_3)$ (Ln = Eu, Gd, Tb, Ho, Yb) structures.

Contrary to the case of $La_3O_2(CN_3)$, for all other lanthanide cations considered in the present study, the refinements of $Ln_3O_2(CN_3)$ (Ln = Eu, Gd, Tb, Ho, Yb) crystal structures suggests a residual electronic density peak at the same position (Fig. SD2.1a).

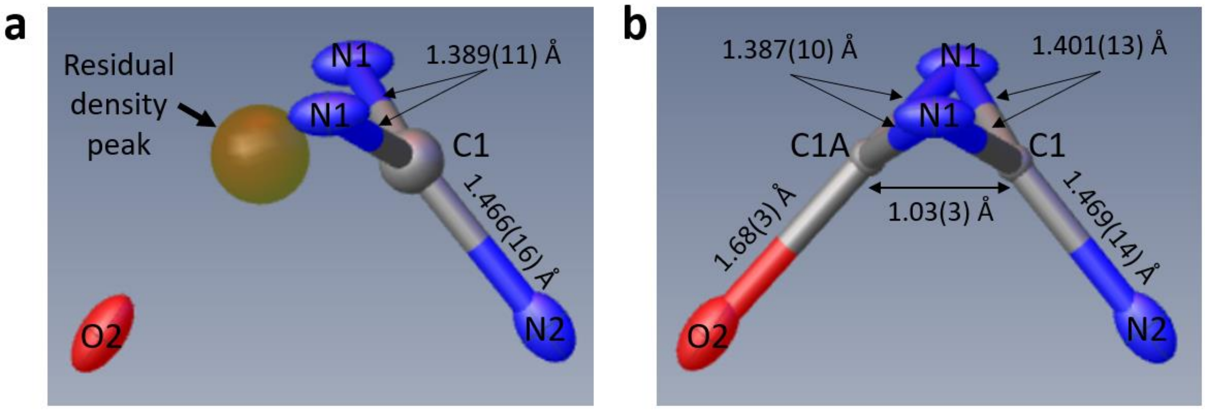

Figure SD2.1. A part of the crystal structure of $Ho_3O_2(CN_3)$ at 1 bar: a) model with a non-split C1 atom and b) model with a split C1 atom (the chemical occupancies of C1 and C1A positions are 0.696(18) and 0.304(18), respectively).

Due to a too short distance from the C1 atom to this residual electronic density peak, as well as insufficient electron density, the simultaneous presence of two C atoms with full occupancy is unreasonable. Therefore, we considered the splitting of C1 between two crystallographic positions (Fig. SD2.1b). This model has a frustrated carbon atom between two CN_3 and CN_2O planar (or almost planar) trigonal coordination environments. The R_1 value drops down compared to the model with a non-split C1 atom (see Tables S6-S10, S12, S13). Interestingly, in most cases, the chemical occupancy ratio between the C1 and C1A positions was ~2:1 (see Tables S6-S10, S12, S13). It is important to note that it was not possible to place the C atom in the center of the CN_3O tetrahedra.

Despite the disorder model resulted in a statistically significant[25] improvement of the R_1 agreement factor, it must be pointed out that the C1A-O2 distance is too long for a C-O single bond, and therefore from a crystal-chemical point of view this model is dubious. We also can not exclude that the appearance of this residual density peak is an artifact of the limited quality of single-crystal X-ray diffraction data collected in the diamond anvil cell. Resolving this question requires additional studies.

Supplementary Figures

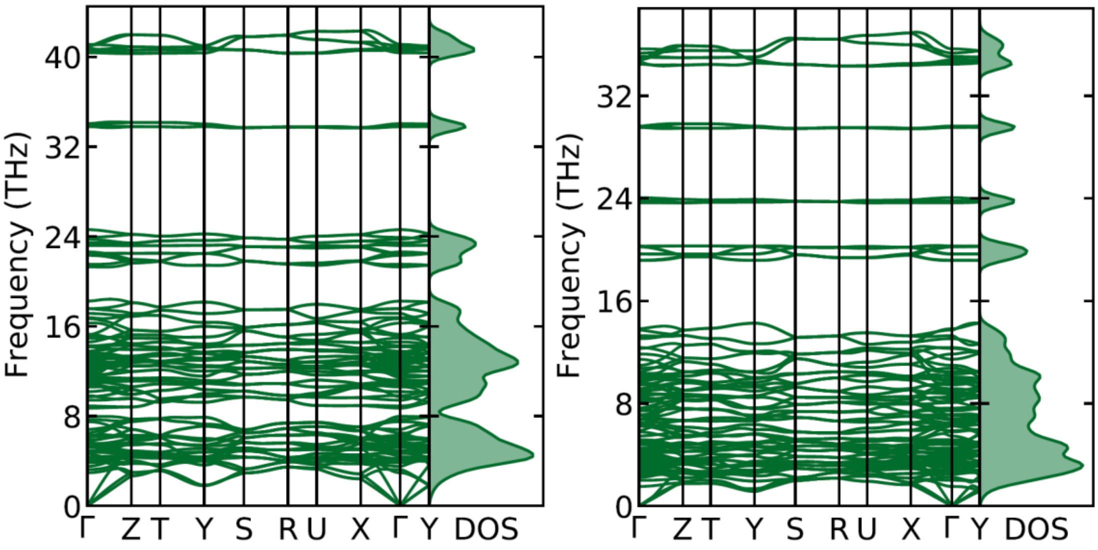

Figure S1. Phonon dispersion curves for $La_3O_2(CN_3)$ along the high symmetry directions in the Brillouin zone and phonon density of states at (a) 54 GPa and (b) ambient pressure.

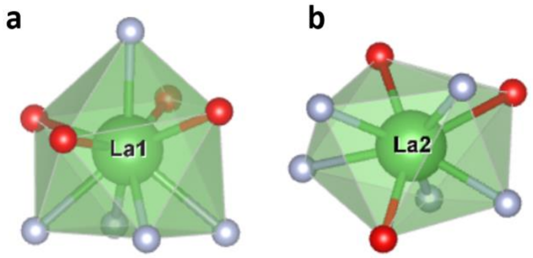

Figure S2. Coordination polyhedra of (a) the La1 atom and (b) the La2 atom in the $La_3O_2(CN_3)$ crystal structure

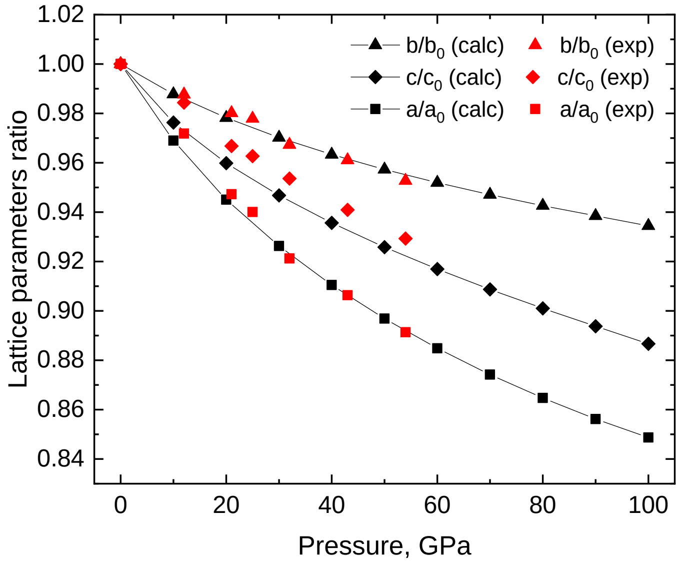

Figure S3. The compressibility of the *a*, *b*, and *c* crystallographic axes of $La_3O_2(CN_3)$. The experimental points are red, and the calculated points are black.

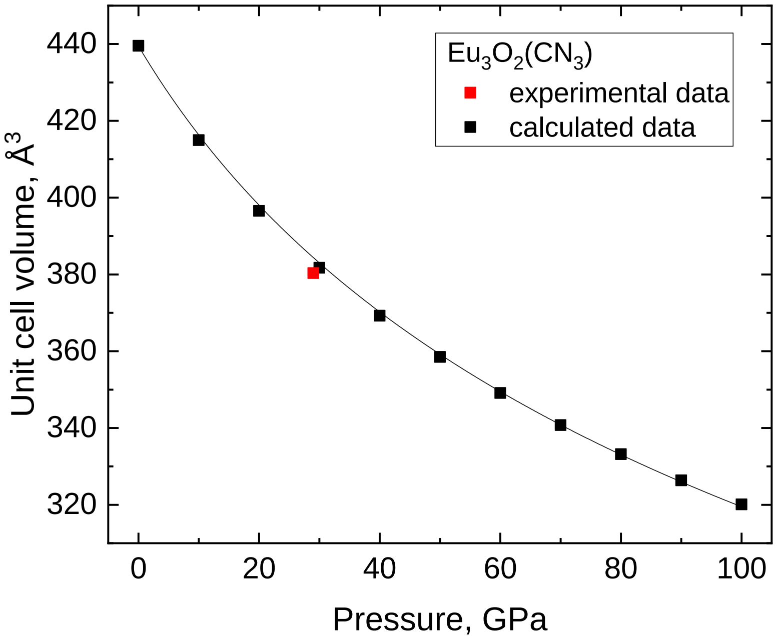

Figure S4. Experimental (red points) and calculated (black points) pressure dependence of the $Eu_3O_2(CN_3)$ unit cell volume. The black curve is the fit of the calculated P-V data using a 2nd order Birch-Murnaghan equation of state, yielding K_0=131.3(9) GPa, K'= 4 (fixed), and V_0=498.16 Å^3 (fixed).

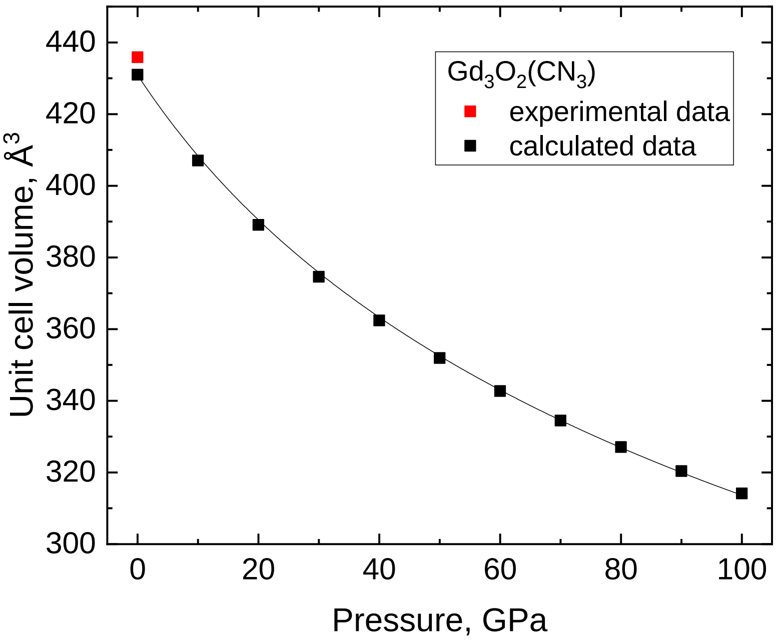

Figure S5. Experimental (red points) and calculated (black points) pressure dependence of the $Gd_3O_2(CN_3)$ unit cell volume. The black curve is the fit of the calculated P-V data using a 2nd order Birch-Murnaghan equation of state, yielding K_0=131.3(9) GPa, K'= 4 (fixed), and V_0=498.16 Å^3 (fixed).

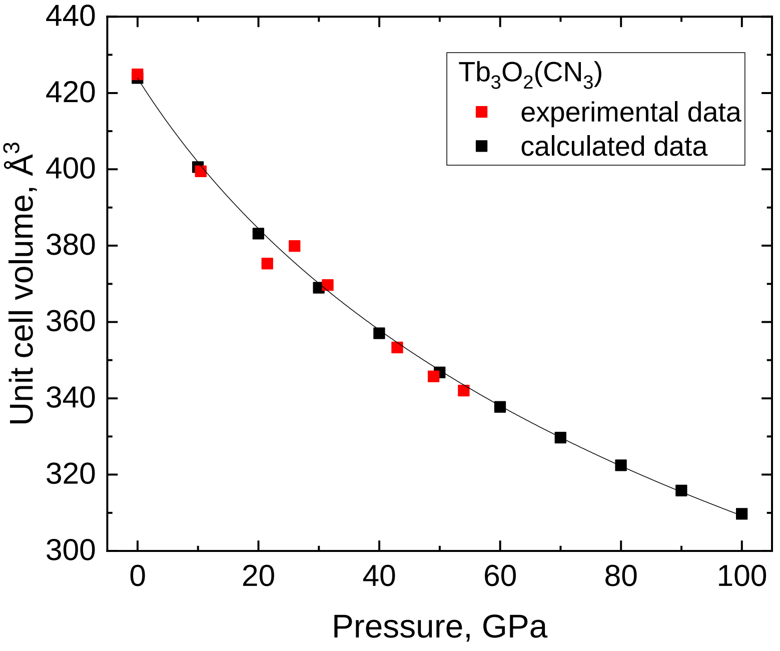

Figure S6. Experimental (red points) and calculated (black points) pressure dependence of the $Tb_3O_2(CN_3)$ unit cell volume. The black curve is the fit of the calculated P-V data using a 2nd order Birch-Murnaghan equation of state, yielding K_0=131.3(9) GPa, K'= 4 (fixed), and V_0=498.16 Å^3 (fixed).

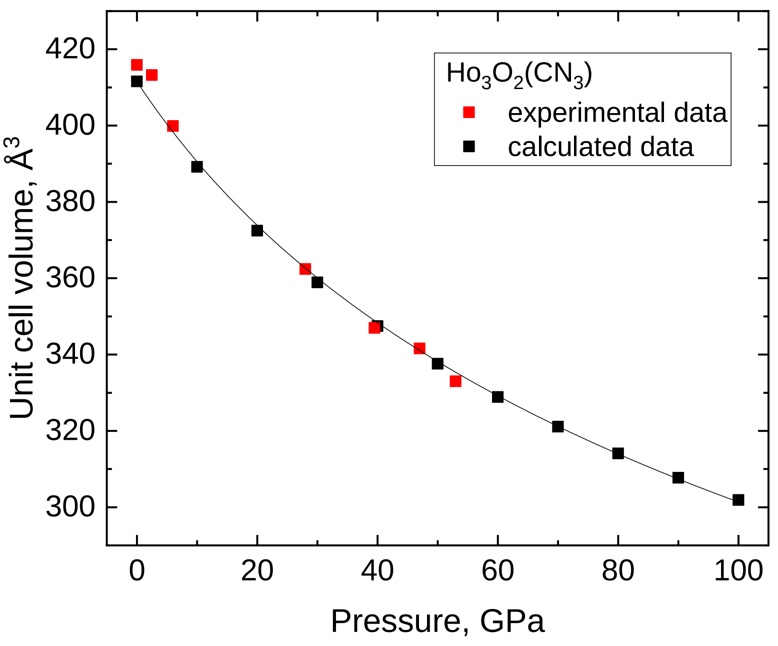

Figure S7. Experimental (red points) and calculated (black points) pressure dependence of the $Ho_3O_2(CN_3)$ unit cell volume. The black curve is the fit of the calculated P-V data using a 2nd order Birch-Murnaghan equation of state, yielding K_0=131.3(9) GPa, K'= 4 (fixed), and V_0=498.16 Å^3 (fixed).

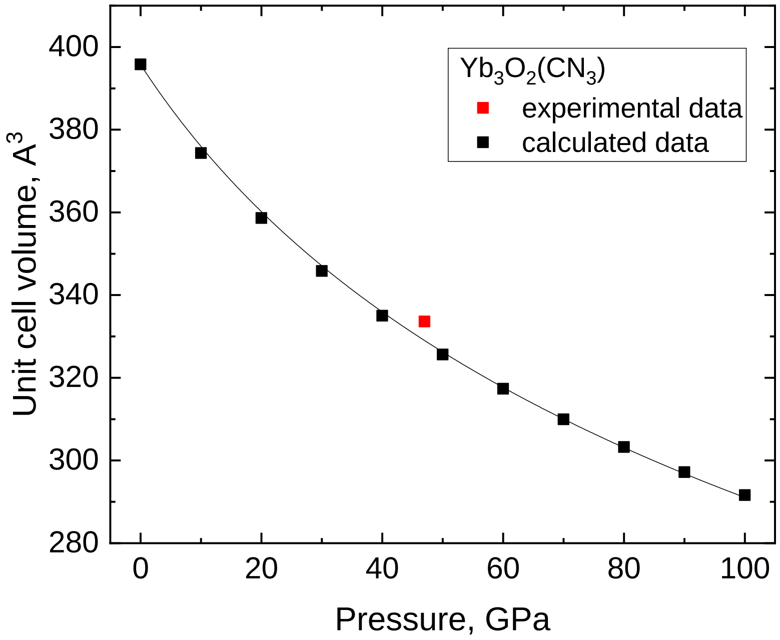

Figure S8. Experimental (red points) and calculated (black points) pressure dependence of the $Yb_3O_2(CN_3)$ unit cell volume. The black curve is the fit of the calculated P-V data using a 2nd order Birch-Murnaghan equation of state, yielding K_0=131.3(9) GPa, K'= 4 (fixed), and V_0=498.16 Å^3 (fixed).

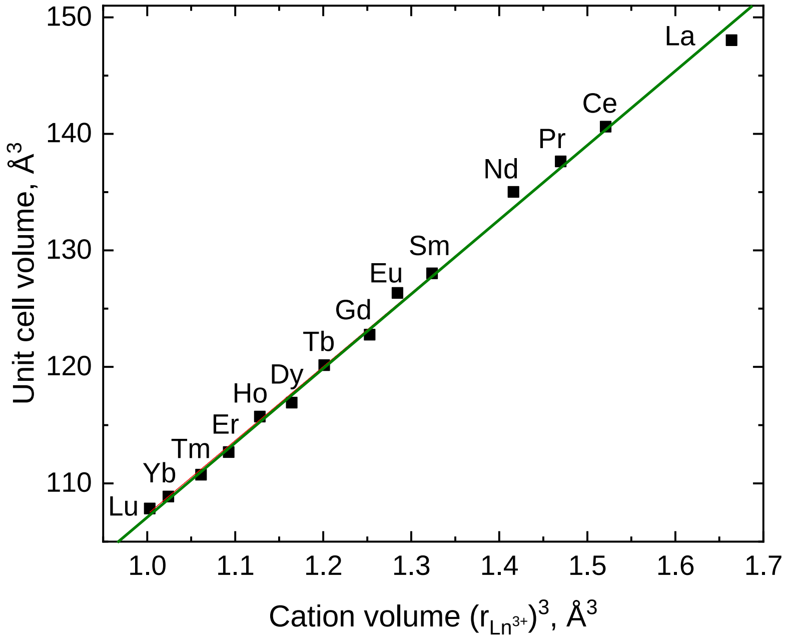

Figure S9. The linear dependence of the unit cell volume of LnN compounds at 1 bar versus the volume of the lanthanide ion (ionic radii are taken from http://abulafia.mt.ic.ac.uk/shannon/ptable.php for CN = 8).

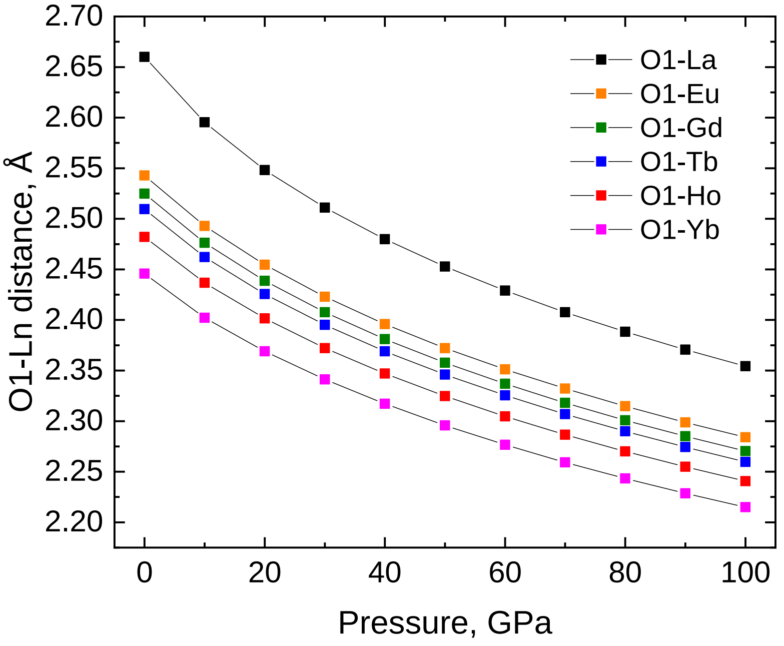

Figure S10. The calculated average O1-Ln bond length within the $O1Ln_6$ octahedra in $Ln_3O_2(CN_3)$ (Ln = La, Eu, Gd, Tb, Ho, Yb) solids, at different pressures.

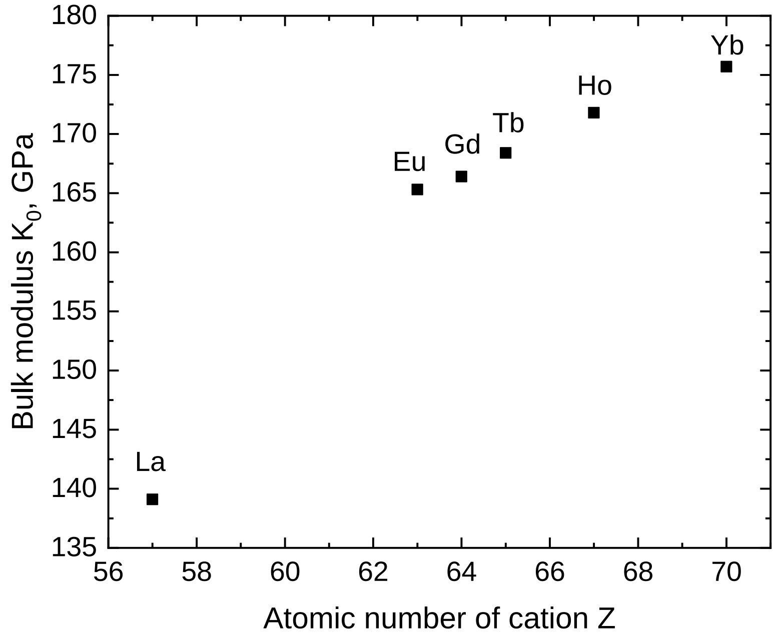

Figure S11. Calculated bulk moduli for $Ln_3O_2(CN_3)$ (Ln = La, Eu, Gd, Tb, Ho, Yb) solids, extracted from 2nd order Birch-Murnaghan equation of state fits.

Supplementary Tables

Table S1. List of the samples investigated in the present study

Sample	Reaction mixture[1]	Pressure, GPa	Temperature, K	Decompression experiment
DAC#1	“La” + N_2	54(1)	2500(500)	Yes
DAC#2	“La” + N_2	25(1)	2500(500)	No
DAC#3	“Tb” + N_2	54(1)	2500(500)	Yes
DAC#4	“Tb” + N_2	26(1)	2500(500)	No
DAC#5	“Gd” + N_2	50(1)	2500(500)	Yes[2]
DAC#6	“Ho” + N_2	53(1)	3000(500)	Yes
DAC#7	“$Eu(N_3)_2$”	29(1)	2000(500)	No
DAC#8	“$Yb(N_3)_2$”	47(1)	2000(500)	No

[1]The quotation marks indicate the nominal composition. In fact, the samples were O-contaminated.

[2]The $Gd_3O_2(CN_3)$ phase was detected only at the last step of the decompression experiment – at ambient conditions. It was not found at higher pressures, presumably because of the presence of other strong-diffracting phases. However, as these phases were no longer visible at ambient conditions due to their decompression, $Gd_3O_2(CN_3)$ could be identified.

Table S2. Structure refinement details of $La_3O_2(CN_3)$ at 54(1) GPa. The full crystallographic dataset was deposited to the CSD under the deposition number 2286277.

**Crystal data**	
Chemical formula	$La_3O_2(CN_3)$
Temperature (K)	293
Pressure (GPa)	54(1)
Mr	502.77
ρ (g/cm^3)	8.566
Crystal system, space group	orthorhombic, *Pnma*
a (Å)	9.2875(7)
b (Å)	6.8259(5)
c (Å)	6.149(2)
V (Å^3)	389.84(14)
Z	4
Radiation type	X-ray, λ = 0.2904 Å
**Data collection**	
No. of measured, independent and observed [I > 2σ(I)] reflections	2095/703/635
R_{int}	3.56%
$(\sin\theta/\lambda)_{max}$ (Å^{-1})	0.999
**Refinement**	
R[F^2 > 3σ(F^2)], wR(F^2), GOF	2.53%, 5.47%, 1.009
data/parameters ratio	703/49
$\Delta\rho_{max}$, $\Delta\rho_{min}$ (e Å^{-3})	1.920, -2.181

**Atomic positions**				
Atom	Wyckoff site	Fractional atomic coordinates		
		x	y	z
La1	8*d*	0.32330(2)	0.01466(3)	0.07787(8)
La2	4*c*	0.47616(3)	0.25	0.60594(11)
O1	4*c*	0.2334(4)	0.25	0.8268(14)
O2	4*c*	0.2943(4)	0.25	0.3672(15)
N1	8*d*	0.0696(3)	0.0736(4)	0.2059(12)
N2	4*c*	0.0081(4)	0.25	0.5113(17)
C1	4*c*	0.0469(5)	0.25	0.305(2)

**Anisotropic displacement parameters**							
Atom	U_{11} (Å^2)	U_{22} (Å^2)	U_{33} (Å^2)	U_{12} (Å^2)	U_{13} (Å^2)	U_{23} (Å^2)	U_{eq} (Å^2)
La1	0.00470(10)	0.00516(9)	0.0099(4)	0.00069(9)	-0.00023(9)	-0.00007(4)	0.00660(14)
La2	0.00537(12)	0.00479(11)	0.0091(6)	0	-0.00011(12)	0	0.00642(18)
O1	0.0048(13)	0.0084(13)	0.003(7)	0	0.0011(16)	0	0.006(2)
O2	0.0094(15)	0.0080(14)	0.012(8)	0	0.0032(19)	0	0.010(2)
N1	0.0065(11)	0.0066(11)	0.005(6)	-0.0005(14)	-0.0018(13)	0.0005(6)	0.0061(17)
N2	0.0048(16)	0.0082(15)	0.007(9)	0	-0.0022(19)	0	0.007(3)
C1	0.009(2)	0.0094(19)	0.020(12)	0	-0.002(3)	0	0.013(4)

Table S3. Structure refinement details of $La_3O_2(CN_3)$ at 25(1) GPa. The full crystallographic dataset was deposited to the CSD under the deposition number 2286275.

Crystal data	
Chemical formula	$La_3O_2(CN_3)$
Temperature (K)	293
Pressure (GPa)	25(1)
Mr	502.77
ρ (g/cm^3)	7.640
Crystal system, space group	orthorhombic, *Pnma*
a (Å)	9.795(2)
b (Å)	7.0062(11)
c (Å)	6.3699(11)
V (Å^3)	437.13(14)
Z	4
Radiation type	X-ray, λ = 0.2904 Å
**Data collection**	
No. of measured, independent and observed [I > 2σ(I)] reflections	1416/594/477
R_{int}	5.53%
$(\sin θ/λ)_{max}$ (Å^{-1})	0.713
**Refinement**	
R[$F^2 > 3σ(F^2)$], wR(F^2), GOF	4.45%, 9.86%, 1.044
data/parameters ratio	594/32
$Δρ_{max}$, $Δρ_{min}$ (e Å^{-3})	2.524, -2.277

Atomic positions

Atom	Wyckoff site	Fractional atomic coordinates		
		x	y	z
La1	8*d*	0.32371(8)	0.01220(9)	0.07030(10)
La2	4*c*	0.47499(11)	0.25	0.60416(15)
O1	4*c*	0.2281(13)	0.25	0.8242(19)
O2	4*c*	0.2956(12)	0.25	0.3636(18)
N1	8*d*	0.0698(11)	0.0742(14)	0.2068(15)
N2	4*c*	0.0082(13)	0.25	0.507(2)
C1	4*c*	0.051(2)	0.25	0.306(3)

Anisotropic displacement parameters

Atom	U_{11} (Å^2)	U_{22} (Å^2)	U_{33} (Å^2)	U_{12} (Å^2)	U_{13} (Å^2)	U_{23} (Å^2)	U_{eq} or U_{iso} (Å^2)
La1	0.0155(4)	0.0061(4)	0.0107(4)	0.00118(19)	0.0001(2)	-0.0006(2)	0.0155(4)
La2	0.0181(6)	0.0061(5)	0.0123(4)	0	-0.0023(4)	0	0.0181(6)
O1							0.015(2)
O2							0.011(2)
N1							0.0132(19)
N2							0.009(2)
C1							0.026(4)

Table S4. Structure refinement details of $La_3O_2(CN_3)$ at 1 bar. The full crystallographic dataset was deposited to the CSD under the deposition number 2286276.

**Crystal data**	
Chemical formula	$La_3O_2(CN_3)$
Temperature (K)	293
Pressure (GPa)	1 bar
Mr	502.77
ρ (g/cm^3)	6.761
Crystal system, space group	orthorhombic, *Pnma*
a (Å)	10.4195(5)
b (Å)	7.1644(3)
c (Å)	6.6169(12)
V (Å^3)	493.95(9)
Z	4
Radiation type	X-ray, λ = 0.2904 Å
**Data collection**	
No. of measured, independent and observed [$I > 2\sigma(I)$] reflections	2185/848/477
R_{int}	4.16%
$(\sin \theta/\lambda)_{max}$ (Å^{-1})	0.862
**Refinement**	
$R[F^2 > 3\sigma(F^2)]$, $wR(F^2)$, GOF	3.16%, 7.79%, 1.020
data/parameters ratio	848/49
$\Delta\rho_{max}$, $\Delta\rho_{min}$ (e Å^{-3})	2.285, -1.830

Atomic positions

Atom	Wyckoff site	Fractional atomic coordinates		
		x	y	z
La1	8*d*	0.32434(2)	0.01033(4)	0.06190(7)
La2	4*c*	0.47527(3)	0.25	0.60030(11)
O1	4*c*	0.2279(5)	0.25	0.8179(15)
O2	4*c*	0.2985(5)	0.25	0.3693(14)
N1	8*d*	0.0693(4)	0.0784(5)	0.2071(12)
N2	4*c*	0.0084(5)	0.25	0.5038(16)
C1	4*c*	0.0506(6)	0.25	0.301(2)

Anisotropic displacement parameters

Atom	U_{11} (Å^2)	U_{22} (Å^2)	U_{33} (Å^2)	U_{12} (Å^2)	U_{13} (Å^2)	U_{23} (Å^2)	U_{eq} (Å^2)
La1	0.00929(14)	0.00728(13)	0.0099(4)	0.00136(11)	-0.00051(9)	-0.00082(6)	0.00884(15)
La2	0.00981(15)	0.00633(15)	0.0092(5)	0	-0.00140(12)	0	0.00845(17)
O1	0.0117(18)	0.0118(17)	0.023(7)	0	-0.006(2)	0	0.016(2)
O2	0.015(2)	0.018(2)	0.007(8)	0	-0.001(2)	0	0.013(2)
N1	0.0236(18)	0.0065(13)	0.004(6)	-0.0018(17)	-0.0036(18)	-0.0009(10)	0.0115(17)
N2	0.0072(19)	0.009(2)	0.021(8)	0	-0.002(2)	0	0.012(2)
C1	0.009(2)	0.013(2)	0.026(10)	0	-0.002(3)	0	0.016(3)

Table S5. Experimentally determined crystallographic data for $La_3O_2(CN_3)$ at 54(1) GPa and 1 bar compared with the corresponding DFT-relaxed structures. Note that pressure was fixed in theoretical simulations, while the volume of the unit cells, lattice parameters and equilibrium state parameters were calculated.

**Pressure**		**54 GPa**		**1 bar**	
**Exp./Calc.**		**Exp.**	**Calc.**	**Exp.**	**Calc.**
**Space group**		*Pnma*	*Pnma*	*Pnma*	*Pnma*
**Volume**		389.84(14) Å^3	389.83 Å^3	493.95(9) Å^3	498.16 Å^3
**Lattice parameters**		a = 9.2875(7) Å<br>b = 6.8259(5) Å<br>c = 6.149(2) Å<br>α = 90º<br>β = 90º<br>γ = 90º	a = 9.3333Å<br>b = 6.8238 Å<br>c = 6.1209 Å<br>α = 90º<br>β = 90º<br>γ = 90º	a = 10.4195(5) Å<br>b = 7.1644(3) Å<br>c = 6.6169(12) Å<br>α = 90º<br>β = 90º<br>γ = 90º	a = 10.4839 Å<br>b = 7.1497Å<br>c = 6.6460Å<br>α = 90º<br>β = 90º<br>γ = 90º
**Atomic positions**	La1 x	0.32330(2)	0.32207	0.32434(2)	0.32382
	La1 y	0.01466(3	0.01489	0.01033(4)	0.00898
	La1 z	0.07787(8)	0.08023	0.06190(7)	0.06165
	La2 x	0.47616(3)	0.47799	0.47527(3)	0.47581
	La2 y	0.25	0.25	0.25	0.25
	La2 z	0.60594(11)	0.60844	0.60030(11)	0.60091
	O1 x	0.2334(4)	0.23533	0.2279(5)	0.22798
	O1 y	0.25	0.25	0.25	0.25
	O1 z	0.8268(14)	0.82501	0.8179(15)	0.81953
	O2 x	0.2943(4)	0.29669	0.2985(5)	0.29808
	O2 y	0.25	0.25	0.25	0.25
	O2 z	0.3672(15)	0.37046	0.3693(14)	0.37844
	N1 x	0.0696(3)	0.06979	0.0693(4)	0.06710
	N1 y	0.0736(4)	0.07400	0.0784(5)	0.07937
	N1 z	0.2059(12)	0.20527	0.2071(12)	0.20813
	N2 x	0.0081(4)	0.00921	0.0084(5)	0.00856
	N2 y	0.25	0.25	0.25	0.25
	N2 z	0.5113(17)	0.50819	0.5038(16)	0.49867
	C1 x	0.0469(5)	0.05542	0.0506(6)	0.04976
	C1 y	0.25	0.25	0.25	0.25
	C1 z	0.305(2)	0.30062	0.301(2)	0.299746

Table S6. Structure refinement details of $Eu_3O_2(CN_3)$ at 29 GPa.

Crystal data						
Chemical formula	$Eu_3O_2(CN_3)$					
Temperature (K)	293					
Pressure (GPa)	29(1)					
Mr	541.92					
ρ (g/cm^3)	9.463					
Crystal system, space group	orthorhombic, *Pnma*					
a (Å)	9.3222(7)					
b (Å)	6.7074(5)					
c (Å)	6.083(6)					
V (Å^3)	380.4(4)					
Z	4					
Radiation type	X-ray, λ = 0.2905Å					
**Data collection**						
No. of measured, independent and observed [I > 2σ(I)] reflections	1434/ 516/ 452					
R_{int}	3.11%					
$(\sin θ/λ)_{max}$ (Å^{-1})	1.071					
**Refinement**						
Structure model	model with a non-split C1 atom			model with split C1 atom		
R[F^2 > 4σ(F^2)], wR(F^2), GOF	3.55%, 9.96%, 1.159			3.32%, 8.90%, 1.074		
data/parameters ratio	516/39			516/39		
$Δρ_{max}$, $Δρ_{min}$ (e Å^{-3})	2.827, -1.831			1.880, -1.753		
**Atomic positions and equivalent isotropic (or isotropic) ADPs for the model with a non-split C1 atom**						
Atom	Wyckoff site	Fractional atomic coordinates			Occupancy	U_{eq} or U_{iso} (Å^2)
		x	y	z		
Eu1	8*d*	0.32594(4)	0.01157(6)	0.0641(2)	1	U_{eq} = 0.0115(5)
Eu2	4*c*	0.47582(6)	0.25	0.5986(3)	1	U_{eq} = 0.0113(6)
O1	4*c*	0.2263(7)	0.25	0.817(3)	1	U_{iso} = 0.014(7)
O2	4*c*	0.2953(10)	0.25	0.354(4)	1	U_{iso} = 0.013(9)
N1	8*d*	0.0767(6)	0.0681(10)	0.193(3)	1	U_{iso} = 0.0119(10)
N2	4*c*	0.0024(9)	0.25	0.528(4)	1	U_{iso} = 0.0082(14)
C1	4*c*	0.0511(18)	0.25	0.318(10)	1	U_{iso} = 0.028(3)
**Atomic positions and equivalent isotropic (or isotropic) ADPs for the model with split C1 atom**						
Atom	Wyckoff site	Fractional atomic coordinates			Occupancy	U_{eq} or U_{iso} (Å^2)
		x	y	z		
Eu1	8*d*	0.32595(4)	0.01154(5)	0.06401(18)	1	U_{eq} = 0.0117(4)
Eu2	4*c*	0.47582(5)	0.25	0.5986(3)	1	U_{eq} = 0.0112(6)
O1	4*c*	0.2267(7)	0.25	0.816(3)	1	U_{iso} = 0.0076(10)
O2	4*c*	0.2953(9)	0.25	0.357(4)	1	U_{iso} = 0.013(8)
N1	8*d*	0.0762(6)	0.0680(9)	0.193(3)	1	U_{iso} = 0.0116(9)
N2	4*c*	0.0028(8)	0.25	0.528(4)	1	U_{iso} = 0.0078(13)
C1	4*c*	0.0504(15)	0.25	0.309(8)	0.67(3)	U_{iso} = 0.013(2)
C1A	4*c*	0.135(3)	0.25	0.261(16)	0.33(3)	U_{iso} = 0.013(2)

Table S7. Structure refinement details of $Gd_3O_2(CN_3)$ at 1 bar.

**Crystal data**						
Chemical formula		$Gd_3O_2(CN_3)$				
Temperature (K)		293				
Pressure (bar)		1				
Mr		557.79				
ρ (g/cm^3)		8.501				
Crystal system, space group		orthorhombic, *Pnma*				
a (Å)		9.939(2)				
b (Å)		6.849(3)				
c (Å)		6.4028(14)				
V (Å^3)		435.8(2)				
Z		4				
Radiation type		X-ray, λ = 0.2904Å				
**Data collection**						
No. of measured, independent and observed [I > 2σ(I)] reflections		1254/ 679 / 399				
R_{int}		10.97 %				
(sin θ/λ)$_{max}$ (Å^{-1})		0.806				
**Refinement**						
Structure model		model with a non-split C1 atom			model with split C1 atom	
R[F^2 > 4σ(F^2)], wR(F^2), GOF		6.86%, 18.22%, 0.950			6.76%, 17.96%, 0.939	
data/parameters ratio		679/32			679/35	
$\Delta\rho_{max}$, $\Delta\rho_{min}$ (e Å^{-3})		4.060, -3.935			3.950, -3.915	
**Atomic positions and equivalent isotropic (or isotropic) ADPs for the model with a non-split C1 atom**						
Atom	Wyckoff site	Fractional atomic coordinates			Occupancy	U_{eq} or U_{iso} (Å^2)
		x	y	z		
Gd1	8*d*	0.32446(11)	0.0087(2)	0.05350(16)	1	U_{eq} = 0.0176(4)
Gd2	4*c*	0.47615(15)	0.25	0.6008(2)	1	U_{eq} = 0.0162(4)
O1	4*c*	0.225(2)	0.25	0.819(3)	1	U_{iso} = 0.021(4)
O2	4*c*	0.294(3)	0.25	0.354(4)	1	U_{iso} = 0.030(6)
N1	8*d*	0.075(2)	0.084(4)	0.196(3)	1	U_{iso} = 0.030(4)
N2	4*c*	0.010(2)	0.25	0.510(4)	1	U_{iso} = 0.018(5)
C1	4c	0.050(3)	0.25	0.300(5)	1	U_{iso} = 0.022(6)
**Atomic positions and equivalent isotropic (or isotropic) ADPs for the model with split C1 atom**						
Atom	Wyckoff site	Fractional atomic coordinates			Occupancy	U_{eq} or U_{iso} (Å^2)
		x	y	z		
Gd1	8*d*	0.32450(11)	0.0086(2)	0.05352(16)	1	U_{eq} = 0.0178(4)
Gd2	4*c*	0.47620(15)	0.25	0.6007(2)	1	U_{eq} = 0.0163(4)
O1	4*c*	0.224(2)	0.25	0.819(3)	1	U_{iso} = 0.021(4)
O2	4*c*	0.294(3)	0.25	0.355(4)	1	U_{iso} = 0.033(6)
N1	8*d*	0.075(2)	0.085(4)	0.198(3)	1	U_{iso} = 0.029(4)
N2	4*c*	0.008(2)	0.25	0.511(4)	1	U_{iso} = 0.019(5)
C1	4*c*	0.049(3)	0.25	0.299(5)	0.72(6)	U_{iso} = 0.008(5)
C1A	4*c*	0.138(8)	0.25	0.238(12)	0.28(6)	U_{iso} = 0.008(5)

Table S8. Structure refinement details of $Tb_3O_2(CN_3)$ at 54 GPa

**Crystal data**		
Chemical formula	$Tb_3O_2(CN_3)$	
Temperature (K)	293	
Pressure (GPa)	54	
Mr	562.80	
ρ (g/cm^3)	10.930	
Crystal system, space group	orthorhombic, *Pnma*	
a (Å)	8.8178(19)	
b (Å)	6.564(6)	
c (Å)	5.909(2)	
V (Å^3)	342.0(3)	
Z	4	
Radiation type	X-ray, λ = 0.29521Å	
**Data collection**		
No. of measured, independent and observed [I > 2σ(I)] reflections	878/ 426 / 379	
R_{int}	1.57%	
$(\sin θ/λ)_{max}$ (Å^{-1})	0.890	
**Refinement**		
Structure model	model with a non-split C1 atom	model with split C1 atom
R[F^2 > 4σ(F^2)], wR(F^2), GOF	3.86%, 10.95%, 1.069	3.66%, 10.40%, 1.070
data/parameters ratio	426/32	426/35
$Δρ_{max}$, $Δρ_{min}$ (e Å^{-3})	3.361, -2.780	3.270, -2.828

**Atomic positions and equivalent isotropic (or isotropic) ADPs for the model with a non-split C1 atom**						
Atom	Wyckoff site	Fractional atomic coordinates			Occupancy	U_{eq} or U_{iso} (Å^2)
		x	y	z		
Tb1	4*c*	0.32452(6)	0.01311(13)	0.07054(8)	1	U_{eq} = 0.0074(3)
Tb2	8*d*	0.47511(9)	0.25	0.60314(11)	1	U_{eq} = 0.0072(3)
O1	4*c*	0.2294(11)	0.25	0.8208(17)	1	U_{iso} = 0.0055(18)
O2	4*c*	0.2904(16)	0.25	0.355(2)	1	U_{iso} = 0.017(2)
N1	8*d*	0.0711(10)	0.070(2)	0.1966(14)	1	U_{iso} = 0.0081(15)
N2	4*c*	0.0034(13)	0.25	0.522(2)	1	U_{iso} = 0.006(2)
C1	4*c*	0.054(2)	0.25	0.298(3)	1	U_{iso} = 0.021(4)
**Atomic positions and equivalent isotropic (or isotropic) ADPs for the model with split C1 atom**						
Atom	Wyckoff site	Fractional atomic coordinates			Occupancy	U_{eq} or U_{iso} (Å^2)
		x	y	z		
Tb1	4*c*	0.32451(5)	0.01314(12)	0.07054(7)	1	U_{eq} = 0.0074(2)
Tb2	8*d*	0.47512(8)	0.25	0.60313(10)	1	U_{eq} = 0.0072(3)
O1	4*c*	0.2294(10)	0.25	0.8211(16)	1	U_{iso} = 0.0061(17)
O2	4*c*	0.2915(15)	0.25	0.356(2)	1	U_{iso} = 0.017(2)
N1	8*d*	0.0704(9)	0.071(2)	0.1973(13)	1	U_{iso} = 0.0080(14)
N2	4*c*	0.0032(12)	0.25	0.5225(19)	1	U_{iso} = 0.006(2)
C1	4*c*	0.053(2)	0.25	0.300(3)	0.68(4)	U_{iso} = 0.006(3)
C1A	4*c*	0.137(5)	0.25	0.265(6)	0.32(4)	U_{iso} = 0.006(3)

Table S9. Structure refinement details of $Tb_3O_2(CN_3)$ at 26 GPa.

Crystal data		
Chemical formula	$Tb_3O_2(CN_3)$	
Temperature (K)	293	
Pressure (GPa)	26	
Mr	562.80	
ρ (g/cm^3)	9.840	
Crystal system, space group	orthorhombic, *Pnma*	
a (Å)	9.399(9)	
b (Å)	6.6399(10)	
c (Å)	6.0873(6)	
V (Å^3)	379.9(4)	
Z	4	
Radiation type	X-ray, λ = 0.41015Å	
**Data collection**		
No. of measured, independent and observed [I > 2σ(I)] reflections	1112 / 382 / 297	
R_{int}	4.53%	
$(\sin θ/λ)_{max}$ (Å^{-1})	0.906	
**Refinement**		
Structure model	model with a non-split C1 atom	model with split C1 atom
R[F^2 > 4σ(F^2)], wR(F^2), GOF	4.32%, 11.09 %, 1.063	4.27%, 11.19%, 1.024
data/parameters ratio	382/32	492/35
$Δρ_{max}$, $Δρ_{min}$ (e Å^{-3})	2.635, -2.405	2.704, -2.198

Atomic positions and equivalent isotropic (or isotropic) ADPs for the model with a non-split C1 atom

Atom	Wyckoff site	Fractional atomic coordinates			Occupancy	U_{eq} or U_{iso} (Å^2)
		x	y	z		
Tb1	4*c*	0.3232(2)	0.01037(11)	0.06292(13)	1	U_{eq} = 0.0139(8)
Tb2	8*d*	0.4759(3)	0.25	0.60687(16)	1	U_{eq} = 0.0149(11)
O1	4*c*	0.222(5)	0.25	0.814(3)	1	U_{iso} = 0.020(4)
O2	4*c*	0.301(5)	0.25	0.357(3)	1	U_{iso} = 0.016(3)
N1	8*d*	0.072(3)	0.0714(17)	0.1960(17)	1	U_{iso} = 0.008(2)
N2	4*c*	-0.001(5)	0.25	0.513(3)	1	U_{iso} = 0.014(4)
C1	4c	0.056(6)	0.25	0.298(3)	1	U_{iso} = 0.015(4)

Atomic positions and equivalent isotropic (or isotropic) ADPs for the model with split C1 atom

Atom	Wyckoff site	Fractional atomic coordinates			Occupancy	U_{eq} or U_{iso} (Å^2)
		x	y	z		
Tb1	4*c*	0.3233(2)	0.01040(11)	0.06291(13)	1	U_{eq} = 0.0137(8)
Tb2	8*d*	0.4759(3)	0.25	0.60689(16)	1	U_{eq} = 0.0152(11)
O1	4*c*	0.223(5)	0.25	0.814(3)	1	U_{iso} = 0.021(3)
O2	4*c*	0.301(4)	0.25	0.357(3)	1	U_{iso} = 0.016(3)
N1	8*d*	0.072(3)	0.0713(17)	0.1957(17)	1	U_{iso} = 0.009(2)
N2	4*c*	0.000(4)	0.25	0.514(3)	1	U_{iso} = 0.013(3)
C1	4*c*	0.051(8)	0.25	0.305(5)	0.73(7)	U_{iso} = 0.003(4)
C1A	4*c*	0.13(2)	0.25	0.253(13)	0.27(7)	U_{iso} = 0.003(4)

Table S10. Structure refinement details of $Tb_3O_2(CN_3)$ at 1 bar.

**Crystal data**		
Chemical formula	$Tb_3O_2(CN_3)$	
Temperature (K)	293	
Pressure (bar)	1	
Mr	562.80	
ρ (g/cm^3)	8.798	
Crystal system, space group	orthorhombic, *Pnma*	
a (Å)	9.8390(4)	
b (Å)	6.8043(8)	
c (Å)	6.3464(4)	
V (Å^3)	424.88(6)	
Z	4	
Radiation type	X-ray, λ = 0.41004Å	
**Data collection**		
No. of measured, independent and observed [I > 2σ(I)] reflections	846/ 492 / 384	
R_{int}	2.14%	
$(\sin θ/λ)_{max}$ (Å^{-1})	0.769	
**Refinement**		
Structure model	model with a non-split C1 atom	model with split C1 atom
R[F^2 > 4σ(F^2)], wR(F^2), GOF	4.50%, 12.03%, 1.027	4.27%, 11.19%, 1.024
data/parameters ratio	492/32	492/35
$Δρ_{max}$, $Δρ_{min}$ (e Å^{-3})	2.811, -2.523	2.704, -2.198

**Atomic positions and equivalent isotropic (or isotropic) ADPs for the model with a non-split C1 atom**						
Atom	Wyckoff site	Fractional atomic coordinates			Occupancy	U_{eq} or U_{iso} (Å^2)
		x	y	z		
Tb1	4*c*	0.32343(7)	0.00880(12)	0.05353(13)	1	U_{eq} = 0.0189(3)
Tb2	8*d*	0.47683(10)	0.25	0.60061(15)	1	U_{eq} = 0.0164(3)
O1	4*c*	0.2229(15)	0.25	0.813(3)	1	U_{iso} = 0.021(3)
O2	4*c*	0.2992(18)	0.25	0.359(3)	1	U_{iso} = 0.030(4)
N1	8*d*	0.0785(13)	0.071(2)	0.194(2)	1	U_{iso} = 0.024(3)
N2	4*c*	0.0012(16)	0.25	0.510(3)	1	U_{iso} = 0.024(4)
C1	4c	0.052(3)	0.25	0.289(5)	1	U_{iso} = 0.035(6)

**Atomic positions and equivalent isotropic (or isotropic) ADPs for the model with split C1 atom**						
Atom	Wyckoff site	Fractional atomic coordinates			Occupancy	U_{eq} or U_{iso} (Å^2)
		x	y	z		
Tb1	4*c*	0.32342(7)	0.00878(11)	0.05348(12)	1	U_{eq} = 0.0189(3)
Tb2	8*d*	0.47685(10)	0.25	0.60064(15)	1	U_{eq} = 0.0165(3)
O1	4*c*	0.2233(14)	0.25	0.813(2)	1	U_{iso} = 0.021(3)
O2	4*c*	0.3002(16)	0.25	0.357(3)	1	U_{iso} = 0.027(3)
N1	8*d*	0.0779(12)	0.074(2)	0.194(2)	1	U_{iso} = 0.023(2)
N2	4*c*	0.0010(16)	0.25	0.510(3)	1	U_{iso} = 0.024(4)
C1	4*c*	0.050(3)	0.25	0.289(4)	0.68(4)	U_{iso} = 0.014(4)
C1A	4*c*	0.147(5)	0.25	0.224(9)	0.32(4)	U_{iso} = 0.014(4)

Table S11. Structure refinement details of $Ho_3O_2(CN_3)$ at 53 GPa.

Crystal data						
Chemical formula		$Ho_3O_2(CN_3)$				
Temperature (K)		293				
Pressure (GPa)		53				
Mr		580.83				
ρ (g/cm^3)		11.586				
Crystal system, space group		orthorhombic, *Pnma*				
a (Å)		8.7617(19)				
b (Å)		6.487(2)				
c (Å)		5.8586(7)				
V (Å^3)		333.00(14)				
Z		4				
Radiation type		X-ray, λ = 0.2901Å				
**Data collection**						
No. of measured, independent and observed [I > 2σ(I)] reflections		1512 / 662 / 459				
R_{int}		2.88 %				
$(\sin θ/λ)_{max}$ (Å^{-1})		1.038				
**Refinement**						
Structure model		model with a non-split C1 atom				
R[F^2 > 4σ(F^2)], wR(F^2), GOF		3.85%, 9.78%, 1.081				
data/parameters ratio		662/32				
$Δρ_{max}$, $Δρ_{min}$ (e Å^{-3})		5.384, -4.830				
**Atomic positions and equivalent isotropic (or isotropic) ADPs for the model with a non-split C1 atom**						
Atom	Wyckoff site	Fractional atomic coordinates			Occupancy	U_{eq} or U_{iso} (Å^2)
		x	y	z		
Ho1	4*c*	0.32419(7)	0.01310(12)	0.07024(8)	1	U_{eq} = 0.00780(18)
Ho2	8*d*	0.47499(10)	0.25	0.60337(13)	1	U_{eq} = 0.00704(14)
O1	4*c*	0.2264(17)	0.25	0.819(2)	1	U_{iso} = 0.012(2)
O2	4*c*	0.2920(18)	0.25	0.353(2)	1	U_{iso} = 0.016(3)
N1	8*d*	0.0699(12)	0.067(2)	0.1994(15)	1	U_{iso} = 0.009(2)
N2	4*c*	0.0034(18)	0.25	0.520(2)	1	U_{iso} = 0.0072(15)
C1	4c	0.056(2)	0.25	0.290(5)	1	U_{iso} = 0.033(6)

Table S12. Structure refinement details of $Ho_3O_2(CN_3)$ at 1 bar.

**Crystal data**		
Chemical formula	$Ho_3O_2(CN_3)$	
Temperature (K)	293	
Pressure (bar)	1	
Mr	580.83	
ρ (g/cm^3)	9.276	
Crystal system, space group	orthorhombic, *Pnma*	
a (Å)	9.7962(5)	
b (Å)	6.7318(8)	
c (Å)	6.3066(3)	
V (Å^3)	415.90(6)	
Z	4	
Radiation type	X-ray, λ = 0.2905Å	
**Data collection**		
No. of measured, independent and observed [I > 2σ(I)] reflections	2153/ 863 / 697	
R_{int}	1.96%	
$(\sin θ/λ)_{max}$ (Å^{-1})	0.908	
**Refinement**		
Structure model	model with a non-split C1 atom	model with split C1 atom
R[F^2 > 4σ(F^2)], wR(F^2), GOF	2.82%, 7.96%, 1.083	2.57%, 6.97%, 1.063
data/parameters ratio	863/46	863/49
$Δρ_{max}$, $Δρ_{min}$ (e Å^{-3})	4.059, -2.045	2.934, -2.135

**Atomic positions and equivalent isotropic (or isotropic) ADPs for the model with a non-split C1 atom**						
Atom	Wyckoff site	Fractional atomic coordinates			Occupancy	U_{eq} or U_{iso} (Å^2)
		x	y	z		
Ho1	4*c*	0.32204(3)	0.00820(7)	0.05145(5)	1	U_{eq} = 0.01184(11)
Ho2	8*d*	0.47778(4)	0.25	0.60101(7)	1	U_{eq} = 0.01014(12)
O1	4*c*	0.2233(7)	0.25	0.8094(10)	1	U_{eq} = 0.0144(18)
O2	4*c*	0.2974(9)	0.25	0.3562(14)	1	U_{eq} = 0.025(3)
N1	8*d*	0.0793(7)	0.0711(15)	0.1922(9)	1	U_{eq} = 0.0184(18)
N2	4*c*	0.0025(8)	0.25	0.5127(18)	1	U_{eq} = 0.018(2)
C1	4*c*	0.0505(11)	0.25	0.2925(17)	1	U_{iso} = 0.023(3)

**Atomic positions and equivalent isotropic (or isotropic) ADPs for the model with split C1 atom**						
Atom	Wyckoff site	Fractional atomic coordinates			Occupancy	U_{eq} or U_{iso} (Å^2)
		x	y	z		
Ho1	4*c*	0.32205(3)	0.00822(6)	0.05145(4)	1	U_{eq} = 0.01181(10)
Ho2	8*d*	0.47778(4)	0.25	0.60102(6)	1	U_{eq} = 0.01008(11)
O1	4*c*	0.2232(6)	0.25	0.8100(9)	1	U_{eq} = 0.0145(16)
O2	4*c*	0.2982(7)	0.25	0.3554(11)	1	U_{eq} = 0.023(2)
N1	8*d*	0.0781(6)	0.0714(13)	0.1920(8)	1	U_{eq} = 0.0186(16)
N2	4*c*	0.0022(6)	0.25	0.5132(16)	1	U_{eq} = 0.018(2)
C1	4*c*	0.0496(9)	0.25	0.2922(15)	0.696(18)	U_{iso} = 0.0076(14)
C1A	4*c*	0.147(2)	0.25	0.231(3)	0.304(18)	U_{iso} = 0.0076(14)

Table S13. Structure refinement details of $Yb_3O_2(CN_3)$ at 47 GPa.

Crystal data						
Chemical formula		$Yb_3O_2(CN_3)$				
Temperature (K)		293				
Pressure (GPa)		47(1)				
Mr		605.16				
ρ (g/cm^3)		12.048				
Crystal system, space group		orthorhombic, *Pnma*				
a (Å)		8.8785(12)				
b (Å)		6.4071(5)				
c (Å)		5.8647(19)				
V (Å^3)		333.62(12)				
Z		4				
Radiation type		X-ray, λ = 0.2905Å				
**Data collection**						
No. of measured, independent and observed [I > 2σ(I)] reflections		1333/ 655 / 573				
R_{int}		1.51%				
$(\sin \theta/\lambda)_{max}$ (Å^{-1})		1.060				
**Refinement**						
Structure model		model with non-split C1 atom			model with split C1 atom	
R[F^2 > 4σ(F^2)], wR(F^2), GOF		2.75%, 7.06%, 1.115			2.41%, 5.87%, 1.111	
data/parameters ratio		655/39			651/45	
$\Delta\rho_{max}$, $\Delta\rho_{min}$ (e Å^{-3})		6.111, -3.163			2.416, -2.692	
**Atomic positions and equivalent isotropic (or isotropic) ADPs for the model with a non-split C1 atom**						
Atom	Wyckoff site	Fractional atomic coordinates			Occupancy	U_{eq} or U_{iso} (Å^2)
		x	y	z		
Yb1	8*d*	0.32577(4)	0.01113(4)	0.06362(8)	1	U_{eq} = 0.00702(12)
Yb2	4*c*	0.47536(5)	0.25	0.59993(11)	1	U_{eq} = 0.00647(14)
O1	4*c*	0.2244(8)	0.25	0.8170(19)	1	U_{eq} = 0.0063(17)
O2	4*c*	0.2949(12)	0.25	0.3540(3)	1	U_{eq} = 0.015(3)
N1	8*d*	0.0792(6)	0.0647(8)	0.1923(15)	1	U_{iso} = 0.0064(8)
N2	4*c*	0.0017(9)	0.25	0.526(2)	1	U_{iso} = 0.0078(13)
C1	4*c*	0.064(3)	0.25	0.308(7)	1	U_{iso} = 0.047(5)
**Atomic positions and equivalent isotropic (or isotropic) ADPs for the model with split C1 atom**						
Atom	Wyckoff site	Fractional atomic coordinates			Occupancy	U_{eq} or U_{iso} (Å^2)
		x	y	z		
Yb1	8*d*	0.32577(4)	0.01109(4)	0.06362(8)	1	U_{eq} = 0.00701(10)
Yb2	4*c*	0.47536(5)	0.25	0.60002(11)	1	U_{eq} = 0.00633(12)
O1	4*c*	0.2233(8)	0.25	0.8192(19)	1	U_{eq} = 0.0072(15)
O2	4*c*	0.2952(12)	0.25	0.3538(3)	1	U_{eq} = 0.015(2)
N1	8*d*	0.0791(6)	0.0653(8)	0.1926(15)	1	U_{eq} = 0.0066(7)
N2	4*c*	0.0016(9)	0.25	0.526(2)	1	U_{eq} = 0.011(2)
C1	4*c*	0.0556(3)	0.25	0.291(7)	0.54(3)	U_{iso} = 0.0081(17)
C1A	4*c*	0.1405(4)	0.25	0.251(8)	0.46(3)	U_{iso} = 0.0081(17)

Table S14. Parameters of the Birch-Murnaghan equation of state fit of $Ln_3O_2(CN_3)$ (Ln = La, Eu, Gd, Tb, Ho, Yb) DFT-relaxed structures

Compound	V_0, Å^3	Parameters of Birch-Murnaghan equation of state fit			
		2nd order		3rd order	
		K_0, GPa	K'	K_0, GPa	K'
$La_3O_2(CN_3)$	498.161 (fixed)	139.1(5)	4 (fixed)	131.3(9)	4.36(4)
$Eu_3O_2(CN_3)$	439.557 (fixed)	165.3(6)	4 (fixed)	156.0(5)	4.41(2)
$Gd_3O_2(CN_3)$	430.982 (fixed)	166.4(5)	4 (fixed)	157.8(6)	4.37(3)
$Tb_3O_2(CN_3)$	423.928 (fixed)	168.4(5)	4 (fixed)	159.8(7)	4.37(3)
$Ho_3O_2(CN_3)$	411.558 (fixed)	171.8(6)	4 (fixed)	162.5(7)	4.40(3)
$Yb_3O_2(CN_3)$	395.793 (fixed)	175.7(7)	4 (fixed)	165.0(9)	4.46(4)

References

[1] I. Kantor, V. Prakapenka, A. Kantor, P. Dera, A. Kurnosov, S. Sinogeikin, N. Dubrovinskaia, L. Dubrovinsky, *Rev. Sci. Instrum.* 2012, *83*, 125102.

[2] R. Boehler, *Rev. Sci. Instrum.* 2006, *77*, 2004–2007.

[3] A. Kurnosov, I. Kantor, T. Boffa-Ballaran, S. Lindhardt, L. Dubrovinsky, A. Kuznetsov, B. H. Zehnder, *Rev. Sci. Instrum.* 2008, *79*, 045110.

[4] G. Aprilis, C. Strohm, I. Kupenko, S. Linhardt, A. Laskin, D. M. Vasiukov, V. Cerantola, E. G. Koemets, C. McCammon, A. Kurnosov, A. I. Chumakov, R. Rüffer, N. Dubrovinskaia, L. Dubrovinsky, *Rev. Sci. Instrum.* 2017, *88*, 084501.

[5] T. Fedotenko, L. Dubrovinsky, G. Aprilis, E. Koemets, A. Snigirev, I. Snigireva, A. Barannikov, P. Ershov, F. Cova, M. Hanfland, N. Dubrovinskaia, *Rev. Sci. Instrum.* 2019, *90*, 104501.

[6] Y. Akahama, H. Kawamura, *J. Appl. Phys.* 2006, *100*, 043516.

[7] S. Anzellini, A. Dewaele, F. Occelli, P. Loubeyre, M. Mezouar, *J. Appl. Phys.* 2014, *115*, 043511.

[8] *Rigaku Oxford Diffraction, CrysAlisPro Softw. Syst. (2015).* 2015, DOI 10.1063/1.2372734.

[9] A. Aslandukov, M. Aslandukov, N. Dubrovinskaia, L. Dubrovinsky, *J. Appl. Crystallogr.* 2022, *55*, 1383–1391.

[10] O. V. Dolomanov, L. J. Bourhis, R. J. Gildea, J. A. K. Howard, H. Puschmann, *J. Appl. Crystallogr.* 2009, *42*, 339–341.

[11] V. Petrícek, M. Dušek, L. Palatinus, *Zeitschrift fur Krist.* 2014, *229*, 345–352.

[12] G. M. Sheldrick, *Acta Crystallogr. Sect. C Struct. Chem.* 2015, *71*, 3–8.

[13] K. Momma, F. Izumi, *J. Appl. Crystallogr.* 2011, *44*, 1272–1276.

[14] J. Gonzalez-Platas, M. Alvaro, F. Nestola, R. Angel, *J. Appl. Crystallogr.* 2016, *49*, 1377–1382.

[15] G. Kresse, J. Furthmüller, *Phys. Rev. B - Condens. Matter Mater. Phys.* 1996, *54*, 11169-11186

[16] G. Kresse, D. Joubert, *Phys. Rev. B - Condens. Matter Mater. Phys.* 1999, *59*, 1758–1775.

[17] J. P. Perdew, K. Burke, M. Ernzerhof, *Phys. Rev. Lett.* 1996, *77*, 3865–3868.

[18] A. Togo, I. Tanaka, *Scr. Mater.* 2015, *108*, 1–5.

[19] S. Yu, B. Huang, Q. Zeng, A. R. Oganov, L. Zhang, G. Frapper, *J. Phys. Chem. C* 2017, *121*, 11037–11046.

[20] S. Wei, D. Li, Z. Liu, X. Li, F. Tian, D. Duan, B. Liu, T. Cui, *Phys. Chem. Chem. Phys.* 2017, *19*, 9246–9252.

[21] Y. Chen, X. Cai, H. Wang, H. Wang, H. Wang, *Sci. Rep.* 2018, *8*, 10670.

[22] X. Shi, Z. Yao, B. Liu, *J. Phys. Chem. C* 2020, *124*, 4044–4049.

[23] X. Du, Y. Yao, J. Wang, Q. Yang, G. Yang, *J. Chem. Phys.* 2021, *154*, 054706.

[24] D. Durach, W. Schnick, *Eur. J. Inorg. Chem.* 2015, *2015*, 4095–4100.

[25] W. C. Hamilton, *Acta Crystallogr.* 1965, *18*, 502–510.
